# Investigations on the Synthesis of Chiral Ionic-Liquid-Supported Ligands and Corresponding Transition-Metal Catalysts: Strategy and Experimental Schemes

**DOI:** 10.3390/molecules29235661

**Published:** 2024-11-29

**Authors:** Di Xu, Xin-Ning Wang, Li Wang, Li Dai, Chen Yang

**Affiliations:** 1 School of Environmental and Municipal Engineering, North China University of Water Resources and Electric Power, Zhengzhou 450046, China; xudi@ncwu.edu.cn (D.X.); z20241060618@stu.ncwu.edu.cn (X.-N.W.); yezi6193@126.com (L.W.); 2 Collaborative Innovation Center for Efficient Utilization of Water Resources, North China University of Water Resources and Electric Power, Zhengzhou 450046, China

**Keywords:** ionic liquids, chiral ligands, quaternization, anion exchange, catalyst recycling

## Abstract

Ionic liquids have been utilized in numerous significant applications within the field of chemistry, particularly in organic chemistry, due to their unique physical and chemical properties. In the realm of asymmetric transition-metal-catalyzed transformations, chiral ionic-liquid-supported ligands and their corresponding transition-metal complexes have facilitated these processes in unconventional solvents, especially ionic liquids and water. These innovative reaction systems enable the recycling of transition-metal catalysts while producing optically active organic molecules with comparable or even higher levels of chemo-, regio-, and stereoselectivity compared to their parent catalysts. In this short review, we aim to provide an overview of the structures of chiral ionic-liquid-supported ligands and the synthetic pathways for these ligands and catalysts. Various synthetic methodologies are demonstrated based on the conceptual frameworks of diverse chiral ionic-liquid-supported ligands. We systematically present the structures and comprehensive synthetic pathways of the chiral ionic-liquid-supported ligands and the typical corresponding transition-metal complexes that have been readily applied to asymmetric processes, categorized by their parent ligand framework. Notably, the crucial experimental procedures are delineated in exhaustive detail, with the objective of enhancing comprehension of the pivotal aspects involved in constructing chiral ionic-liquid-tagged ligands and compounds for both scholars and readers. Considering the current limitations of such ligands and catalysts, we conclude with remarks on several potential research directions for future breakthroughs in the synthesis and application of these intriguing ligands.

## 1. Introduction

Ionic liquids (ILs) are low-temperature molten salts composed of cations and anions that remain liquid below 100 °C. The foundational work on organic salts at room temperature was reported in 1914 by Walden. The first generation of ionic liquids, identified in 1948 by Hurley, involved colorless and transparent liquids formed by mixing alkyl pyridine and aluminum chloride [1]. The second generation of ionic liquids, featuring an imidazolium cation that is insensitive to both water and air, was discovered by Wilkes and Zaworotko in 1992 [2]. Nowadays, a wide variety of ionic liquids are noted in the literature for their negligible vapor pressure, excellent chemical and thermal stability, structural tunability, and environmental friendliness [3,4,5,6,7]. 

**Figure 1:**
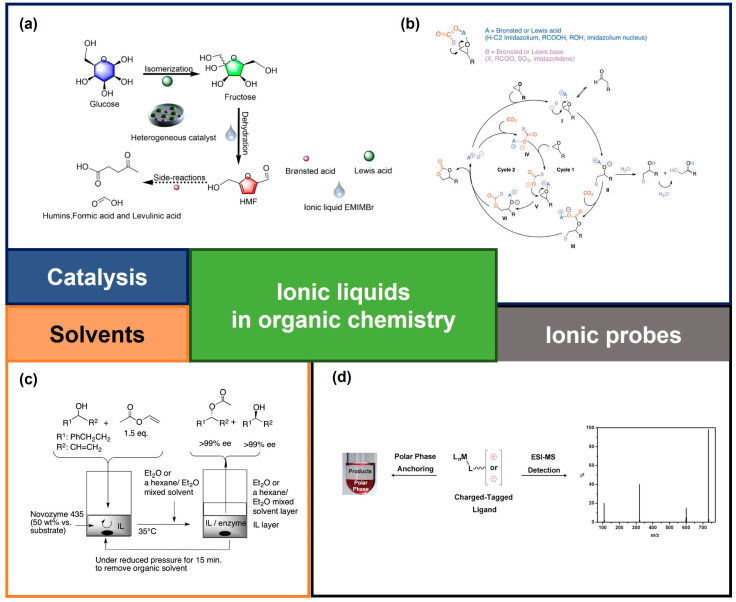
Select examples of ionic liquids utilized in the area of organic chemistry. (**a**) Conversion of glucose to HMF with ionic liquids. Reprinted with permission from Ref. [10]. Copyright 2022, Elsevier B. V.; (**b**) Proposed mechanism of CO_2_ cycloaddition with styrene oxide catalyzed by ionic liquids. Reprinted with permission from Ref. [14]. Copyright 2021, Wiley-VCH GmbH; (**c**) Lipase-catalyzed enantioselective transesterification of in ionic liquids. Reproduced with permission from Ref. [20]. Copyright 2017, American Chemical Society; (**d**) Charge-tagged ligands for polar phase anchoring and ESI-MS detection. Reproduced from Ref. [23] with permission from the Royal Society of Chemistry.

The unique properties of ILs have made them a powerful tool in organic chemistry research, where they have been involved in various organic transformations with significant academic and industrial applications, leading to fascinating results [8,9]. For instance, Li’s group recently investigated methods for producing the important intermediate 5-hydroxymethylfurfural (HMF) from biomass materials to industrial products (Figure 1a) [10]. The process involving ionic liquids for HMF preparation showed outstanding results [11,12,13]. Furthermore, recyclable metal catalysts supported by polymeric ionic liquids, formed from selected ionic liquids and metal salts, effectively catalyzed the conversion of glucose to HMF, maintaining a stable yield over six catalytic cycles, as demonstrated by Chen’s group [11]. In the study of CO_2_ fixation, Yin’s group in 2021 discussed the fixation of CO_2_ into cyclic carbonates by designing ILs as suitable catalysts in both homogeneous and heterogeneous processes (Figure 1b) [14] This review systematically investigated the macro- and microscopic structures of ionic liquids and the preparation of supported ionic liquids on catalytic reaction results [15,16,17,18] and indicated the general principles and future directions for the design of novel IL catalysts. In biocatalytic transformations [19], Itoh in 2017 showcased more than 13 types of enzyme-catalyzed reactions that could be carried out in IL solvents, highlighting the activation of lipase-catalyzed transesterification reactions using IL technology (Figure 1c) [20]. In electrospray ionization mass spectrometry (ESI-MS) experiments [21,22], Dupont cited examples of using charged ligands as ionic probes for detecting reactive intermediates and conducting mechanistic studies of organometallic-catalyzed reactions (Figure 1d) [23]. The insertion of an ionic tag during the detection process ensured that the charge of the intermediate was not induced by the ionization source, solving the problem of detecting “ESI-MS-blind intermediates”.

**Figure 2:**
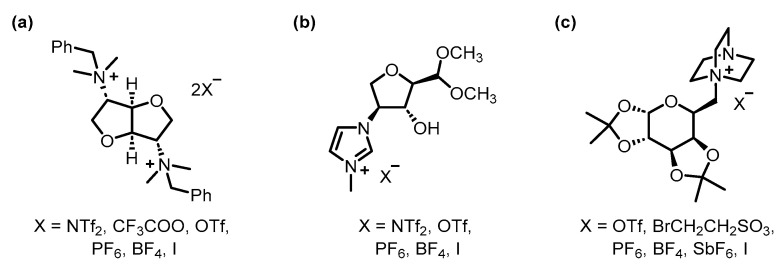
Chiral SILs (CHILs) used in differentiation of racemates in NMR spectroscopy. (**a**) *D*-isomannide derived carbohydrate-based bis(ammonium) chiral ionic liquids; (**b**) *D*-xylose derived carbohydrate chiral ionic liquids; (**c**) *D*-galactose derived carbohydrate chiral ionic liquids.

In the field of organic chemistry, the efficient preparation of chiral substances is a critically important subfield that has garnered significant attention from chemical scientists. The introduction of ionic liquids (ILs) into chiral chemistry has undoubtedly enhanced the ability to synthesize and study chiral compounds, as evidenced by numerous recent research papers and reviews [24,25,26]. For example, over the past decade, a new class of ionic liquids based on natural sugars has been designed and successfully applied to the synthesis of chiral compounds [27]. These sugar-based ionic liquids (SILs), also known as carbohydrate-based ionic liquids (CHILs), have been shown to function as chiral recognition reagents in the differentiation of various racemates in NMR spectroscopy. This application has been presented by the research groups of Malhotra (Figure 2a) [28,29], Reddy (Figure 2b) [30], and Chopra (Figure 2c) [31] respectively.

Additionally, asymmetric synthesis methods, particularly catalytic stereoselective transformations using chiral catalysts, have been extensively applied in both laboratory research and industrial production for the precise synthesis of chiral substances. As a result, a wide variety of chiral catalysts have been synthesized, and their catalytic properties have been verified in corresponding asymmetric reactions. During this period, chiral ionic-liquid-supported ligands and catalysts, which structurally combine highly efficient catalysts with ionic liquids, have been well-designed and synthesized [32]. These catalysts typically achieve comparable or even superior reaction outcomes compared to their unfunctionalized parent catalysts. Intriguingly, the incorporation of ionic liquid fragments into the catalysts enhances their solubility in ionic liquids or polar solvents (such as water), facilitating the production of optically active products through low-cost, environmentally friendly, and sustainable approaches [33]. This advantage often allows the chiral catalysts to be recycled through simple experimental procedures, yielding robust chemical and stereoselective results during the recycling process [7].

Prior to our publication of this manuscript, numerous research groups have focused on the development of chiral ionic-liquid-supported ligands and catalysts [34,35,36,37,38]. Previous studies have discussed various issues related to the application of chiral catalysts and their exceptional catalytic reaction results. However, we strongly believe that practical synthesis methods of chiral ligands and catalysts are key to realizing the corresponding asymmetric transformations. This short review presents a classification of the synthesis methods employed in the preparation of chiral ionic-liquid-supported ligands and catalysts. The specific synthesis methods of the corresponding chiral supported compounds are described based on their parent catalysts. To provide a more comprehensive illustration of the experimental procedures utilized in the synthesis of these compounds, several representative examples are detailed. Furthermore, the potential applications of chiral ionic-liquid-supported catalysts in asymmetric catalytic reaction systems are discussed. We hope that the content of this short review will provide the readers of *Molecules* with a comprehensive understanding of the synthetic methods for chiral ionic-liquid-supported compounds and offer valuable insights for the design and synthesis of novel chiral ionic-liquid-supported ligands, catalysts, and materials.

## 2. Synthesis Strategy for Ionic-Liquid-Supported Chiral Catalysts

The design of chiral ionic-liquid-supported catalysts does not involve fundamentally remodeling the chiral ligands or their corresponding catalysts. Generally, the newly designed chiral supported compounds tend to preserve the parent structure of the ligands or catalysts, which already exhibit good capability in catalytic asymmetric transformations. These structures are then aligned with selected ionic liquid fragments at suitable positions within the parent structure. This approach ensures that the ability of the functionalized catalyst to stereocontrol the enantioselective products in a homogeneous catalytic system is retained as much as possible, while the introduced ionic liquid moiety serves as a functional group.

### 2.1. Covalent Bonding Strategies

**Figure 3:**
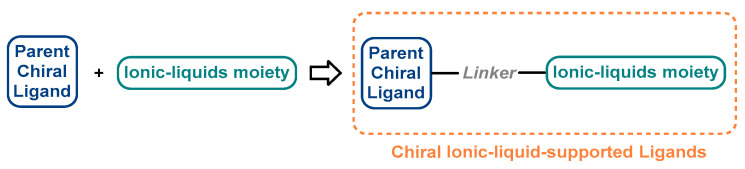
Synthetic strategy on covalent bonding strategies.

The term “covalent bonding strategies” describes one of the typical methodologies for constructing target ionic-liquid-supported molecules (Figure 3). This is achieved by linking a chiral ligand or its corresponding transition-metal catalyst parent to a specific fragment containing an ionic liquid moiety, using a selected linker group (*linker*). Chiral ligands or catalysts obtained through this synthetic strategy typically possess two distinct characteristics: Firstly, the preparation processes of the target compounds are straightforward and yield satisfactory results. The synthesis of chiral ligands and catalysts with different anionic and cationic structural fragments can be easily achieved by modifying the fragment structure of the ionic liquid moiety. Secondly, the ionic liquid groups in chiral ligands are typically situated at a considerable distance from the catalyst’s active center. Consequently, the ionic liquid fragments usually function as auxiliary groups in catalytic processes, enhancing the solubility of the catalyst in ionic liquids or water or facilitating the construction of catalyst recycling processes.

Chemists select an appropriate linker group based on the parent chiral ligands and the ionic-liquid-tagged units. The *linker* is typically represented by groups such as amides (or sulfonamides), imines, phosphites, etc. Common linkers and representative chiral ligands are displayed in Table 1. It should be noted that in Table 1 and the subsequent reaction schemes, an effort has been made to differentiate the components of the chiral ligand structure through the use of varying colors. The backbone structure of the ligand, which typically plays a determining role in the selectivity of the optically active products during asymmetric catalysis, is indicated in blue. The ionic liquid fragment, representing the functional group, is labeled in green. The *linker* that connects these two parts is highlighted in grey. For complex structures involving transition-metals, the catalytic center is represented in orange. Additionally, in certain chiral architectures, the stereoisomer configuration has been tailored to suit the specific requirements of the catalytic process, and these modifications are depicted in purple.

### 2.2. Ionization Strategy (Quaternization Strategy)

In the previously discussed covalent bonding strategy, the chiral ligands and the tagged ionic liquid fragments in catalyst molecules were typically positioned at a considerable distance from the catalytic center. This arrangement meant that the charged ionic liquid fragments had minimal direct influence on the selectivity of the optically active products produced during the catalytic transformation. It is noteworthy, however, that the charge distribution within ionic liquid fragments has been shown to affect the electron density at the catalytic active site, particularly in transition-metal complexes. This, in turn, can influence both the chemical and stereoselectivity of asymmetric catalytic reactions. In contrast to the covalent bonding strategy, ionization strategies offer the advantage of positioning ionic liquid fragments closer to the catalytically active center, thereby amplifying their charge effects on the asymmetric transformation.

Rooted in traditional chiral ligand structures, ionization strategies can be classified into two distinct approaches:

#### 2.2.1. Pre-Cation Approach

**Figure 4:**
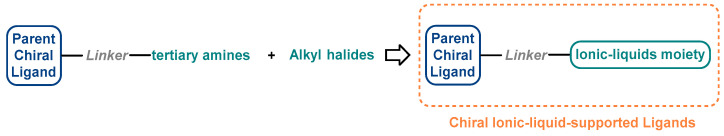
Synthetic strategy on pre-cation approach for chiral ionic-liquid-supported ligands.

In the pre-cation approach, a nitrogen-containing group (e.g., imidazole, pyridine) is retained within the parent structure of the catalyst. This group is then subjected to quaternization with haloalkanes or sulfonate esters, transforming the nitrogen-containing group in the parent structure into a positively charged cationic group. This transformation enables the synthesis of the target compound (Figure 4).

#### 2.2.2. Pre-Anion Approach

**Figure 5:**
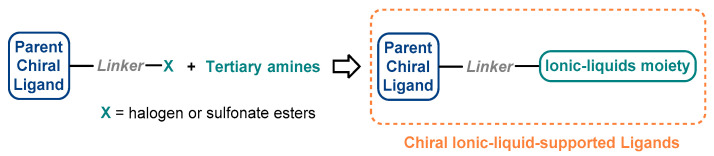
Synthetic strategy on pre-anion approach for chiral ionic-liquid-supported ligands.

Compared to the pre-cation approach, another alternative involves introducing halogen atoms or reactive groups, such as hydroxyl groups, into the structure of conventional organic compounds. Researchers have attempted to incorporate C–X bonds (where X represents halogens with comparatively low bond energies) or sulfonate groups into chiral ligand structures to achieve high ionic selectivity. Subsequently, these ligands undergo quaternization with nitrogen-containing groups (e.g., imidazole, pyridine) to construct novel structures of chiral ionic-liquid-supported ligands (Figure 5).

### 2.3. Anion Exchange

**Figure 6:**
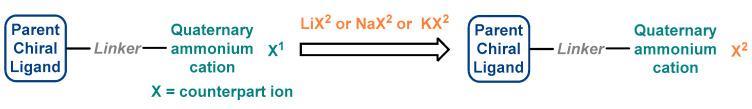
General route of anion exchange of chiral ionic-liquid-supported ligands.

In the prepared chiral ionic-liquid-supported ligands, cationic fragments such as imidazolium, pyridinium, and quaternary ammonium salts were attached to their parent catalyst structure. It is noteworthy that the counterions are more flexible and can be significantly altered through anion exchange (Figure 6). In fact, the results from asymmetric catalytic reactions show that altering the anion structure in ionic-liquid-supported ligands/catalysts can effectively influence the solubility of the catalysts in reaction solvents, as well as impact the product selectivity during the catalytic process. This highlights that changes in the anions of chiral ligands play a decisive role in the successful application of catalysts in asymmetric transformations [39,40,41].

## 3. Synthetic Methods of Chiral Ionic-Liquid-Supported Ligands for Transition-Metal Catalysts

### 3.1. Ferrocene-Type Ligands

Due to their ease of derivatization and unique “planar chirality”, ferrocene analogues have become an important class of chiral ligands in asymmetric transition-metal catalysis [42,43,44,45]. The first report on the synthesis and application of chiral ferrocene-type ligands containing ionic liquid fragments was published by Šebesta in 2007 [46]. Using the ionic liquid [Emim]SO_4_Et as a solvent, the catalytic nucleophilic substitution with Pd-allyl intermediates demonstrated a significant improvement in both the catalytic activity and recoverability of chiral ligands incorporating imidazolium fragments, compared to their parent ligands in Toma’s work [47,48].

**Scheme 1:**
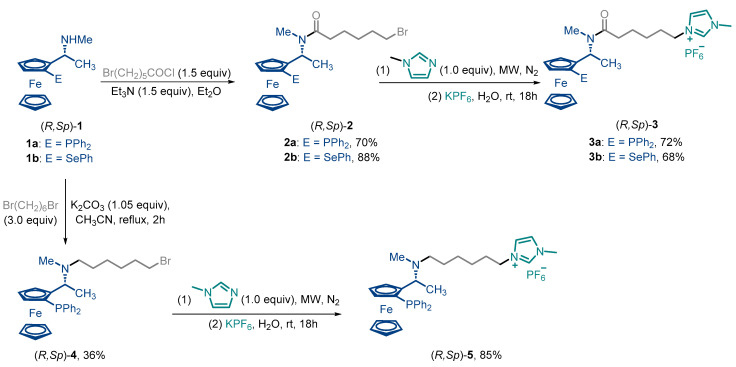
Synthesis of planar chiral imidazolium-tagged ferrocene ligands by Šebesta’s group.

The synthetic approaches for the imidazolium-tagged ferrocenyl ligands are outlined in the following schemes. Šebesta began with the well-established Ugi’s amine as the starting material, which was then transformed into planar chiral ferrocene derivatives through diastereoselective *ortho*-lithiation with BuLi. The reaction was followed by treatment with PPh_2_Cl and (PhSe)_2_, respectively. The *α*-carbon adjacent to the ferrocene moiety was subsequently converted into the desired amines (*R*,*Sp*)-**1** via a stepwise nucleophilic substitution. By reacting (*R*,*Sp*)-**1** with 6-bromohexanoyl chloride or 1,6-dibromohexane, the key intermediates (*R*,*Sp*)-**2** and (*R*,*Sp*)-**4** with the six-carbon linker were successfully obtained. The imidazolium salt compounds were then reacted with brominated ferrocene amines and 1-methylimidazole under microwave irradiation. The bromide anion was exchanged for a non-coordinating hexafluorophosphate in water. The target planar chiral imidazolium-tagged ferrocene ligands (*R*,*Sp*)-**3** and (*R*,*Sp*)-**5** were then obtained in good yields (Scheme 1).

**Scheme 2:**
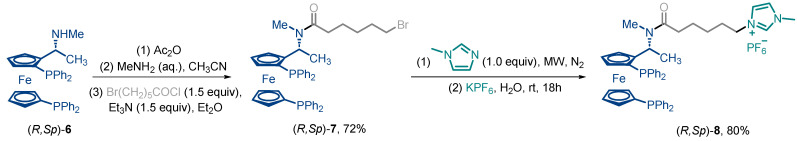
Synthesis of planar chiral imidazolium-tagged bisphosphine ferrocene ligand.

Similarly, the synthesis of bisphosphine ferrocene with an imidazolium fragment was reported in the same work by Šebesta (Scheme 2). Starting with BPPFA, the designed imidazolium-labeled bisphosphine ferrocene ligand (*R*,*Sp*)-**8** was obtained through a sequence of steps: amino conversion (yielding (*R*,*Sp*)-**6**), bridge chain introduction (yielding (*R*,*Sp*)-**8**), followed by salt formation and anion exchange. The overall yield of the ferrocene compound was reported to be 57%.

In the same year, Blaser’s group achieved rhodium-catalyzed enantioselective hydrogenation of functional olefins in biphasic co-solvent/ionic liquid combinations [49]. The introduction of the imidazolium salt unit significantly enhanced the solubility of the ferrocene-type ligands, as well as their corresponding transition-metal complexes, in commonly used ionic liquids such as [bmim]BF_4_. This outstanding feature facilitated catalytic hydrogenation with modified ligands, leading to more robust recycling results.

**Scheme 3:**
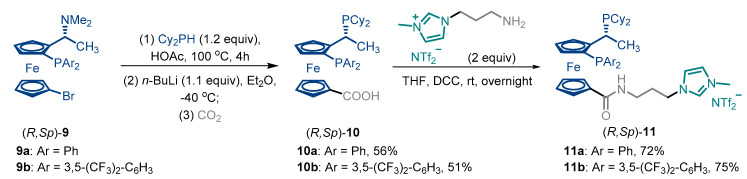
Synthesis of planar chiral imidazolium-tagged bisphosphine ferrocene ligand.

The synthesis of modified Josiphos ligands bearing the imidazolium unit is shown in Scheme 3. The planar chiral 1′-bromo Josiphos ligands (*R*,*Sp*)-**10** were obtained by substituting the amine group of monophosphine ferrocenes (*R*,*Sp*)-**9** with Cy_2_PH in AcOH. The introduction of the imidazolium moiety began with the derivatization of the bromide at the non-substituted Cp ring, which was then converted to a carboxyl acid through a classical Br/Li exchange and reaction with CO_2_. The target functionalized Josiphos-type ligands (*R*,*Sp*)-**11** were finally obtained by DCC-promoted amidation with the selected imidazolium-modified amine.

Another example of chiral ferrocene ligands containing ionic liquid fragments, synthesized from Ugi’s amine, was reported by Zhou’s group in 2010 [50]. The high yields of alkylation products and the excellent stereoselectivity observed in catalytic experiments demonstrated that the designed planar chiral imine-phosphine ferrocene ligands were effective for Pd-catalyzed asymmetric allylic substitution reactions. Furthermore, the catalytic results using quaternary-ammonium-free ligands confirmed that the ionic liquid fragment positively influenced the yield and stereoselectivity of the alkylation products [51].

**Scheme 4:**
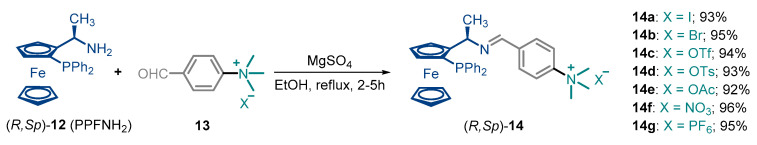
Synthesis of planar chiral quaternary-ammonium-tagged ferrocene ligands.

The reported planar chiral imine-phosphine ferrocene ligands were synthesized based on (*R*)-1-[(*Sp*)-2-(diphenylphosphino)ferrocenyl]ethylamine ((*R*,*Sp*)-**12**, PPFNH_2_), which was reacted with benzaldehyde, readily available, and coupled with a quaternary ammonium fragment in absolute alcohol under argon. Pure products (*R*,*Sp*)-**14** were obtained by recrystallization, yielding 92–96% (Scheme 4). The results for the yields of similar planar chiral imine-phosphine ferrocene ligands (**15**–**18**) are listed in Table 2.

**Scheme 5:**
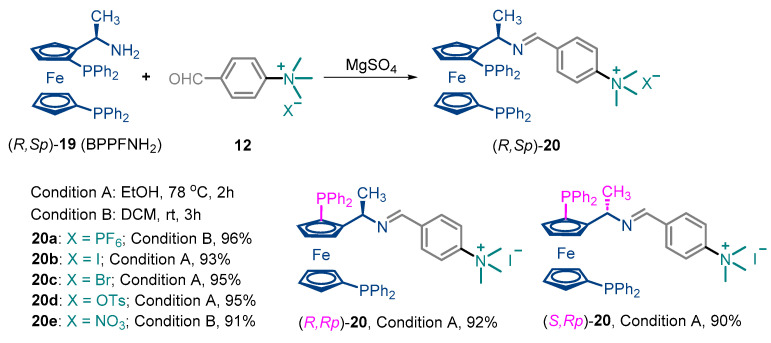
Synthesis of planar chiral quaternary-ammonium-tagged diphosphine ferrocene ligands.

Later, Zhou’s group discussed the synthetic scheme for ionic-liquid-tagged imino-diphosphine ferrocene ligands and their potential application in Pd-catalyzed asymmetric allylic etherification of 1,3-diphenyl-2-propenyl acetate with benzyl alcohols [52]. The reported diphosphine ligands (*R*,*Sp*)-**20** were synthesized from 1-(1′,2-bis-diphenylphosphinyl)ferrocenyl amine ((*R*,*Sp*)-**19**, BPPFNH_2_) following the synthetic route outlined in their previous work. The amine group of (*R*,*Sp*)-**19** was reacted with appropriate aldehydes containing a quaternary ammonium group to form the target ligands in absolute alcohol under an inert atmosphere, yielding the desired planar chiral ferrocenes in a 91–96% yield (Scheme 5). To investigate the influence of the stereochemical structure in the chiral ligands on the stereoselectivity of the Pd-catalyzed asymmetric allylic substitution products, ionic-liquid-supported planar chiral ferrocene ligands with different configurations, (*R*,*Rp*)-**20** and (*S*,*Rp*)-**20**, were also synthesized. A series of ionic liquid fragments were incorporated into the chiral ligands, with BPPFNH_2_ serving as the backbone, as shown in Table 3.

Chiral ligands with a 2-oxazoline ferrocene framework, which have been used as key chiral scaffolds for achieving optically active organic blocks with excellent stereoselectivity [53], have also been selected for the synthesis and application of supported ligands. In 2015, Zhou’s group reported ionic-liquid-supported planar chiral oxazoline-phosphine ferrocene ligands and their successful stereoselective applications in Cu-catalyzed [3+2] 1,3-dipolar cycloaddition reactions [54] and Ru-catalyzed hydrogenation of aromatic ketones [55]. Notably, both the ferrocene-transition-metal catalysts demonstrated successful recycling with stable results.

**Scheme 6:**
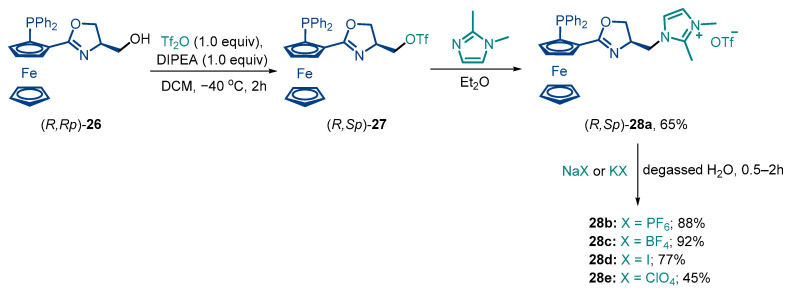
Synthesis of imidazolium-tagged oxazoline phosphine ferrocene ligands.

The synthetic strategy for the ionic-liquid-supported 2-oxazoline phosphine ferrocene ligands is shown in Scheme 6. The experimental procedure for the preparation of the key planar chiral ferrocene compound (*R*,*Rp*)-**26** is described in the following section (see Section 5.1). The hydroxyl group on the chiral side chain was esterified with stoichiometric amounts of trifluoromethanesulfonic anhydride (Tf_2_O) at −40 °C. The crucial but unstable esterification product (*R*,*Sp*)-**27** was purified by flash column chromatography at room temperature and then rapidly quaternized with the selected amines, yielding the ionic-liquid-supported ferrocene ligands (*R*,*Sp*)-**28a**. Anion exchange of the trifluoromethyl sulfonate products with alkali metal salts in degassed water provided the desired ionic-liquid-supported ferrocenes (*R*,*Sp*)-**28** with different anions. Similarly, the *N*-methylpyrrolidinium-tagged ferrocene ligand used in the enantioselective hydrogenation of aromatic ketones[55] was prepared using the method shown in Scheme 6.

### 3.2. Bisoxazolines

Chiral molecules with *C*_2_-symmetry bearing two oxazoline moieties linked by a spacer unit are referred to as bisoxazoline ligands. Since the first report in 1991, hundreds of bisoxazoline-type chiral ligands have been designed and synthesized, playing a prominent role in various asymmetric transition-metal-catalyzed reactions [56]. To further reduce the high cost of synthetic ligands and catalytic processes, the immobilization and recycling of bisoxazolines and their corresponding complexes have already been demonstrated by several research groups.

In 2007, Doherty and Knight combined the bisoxazoline parent structure with an ionic liquid fragment, and the resulting imidazolium-salt-tagged bisoxazoline ligands were applied to Cu(II)-catalyzed Diels–Alder reactions [57]. In contrast to the catalytic results with unfunctionalized ligands, the catalytic reaction proceeded at a much faster rate with the imidazolium-salt-labeled bisoxazoline ligands at a lower catalyst level. Additionally, the catalyst recovery process was successfully completed over 10 cycles under ionic liquid system conditions with negligible catalyst loss.

**Scheme 7:**
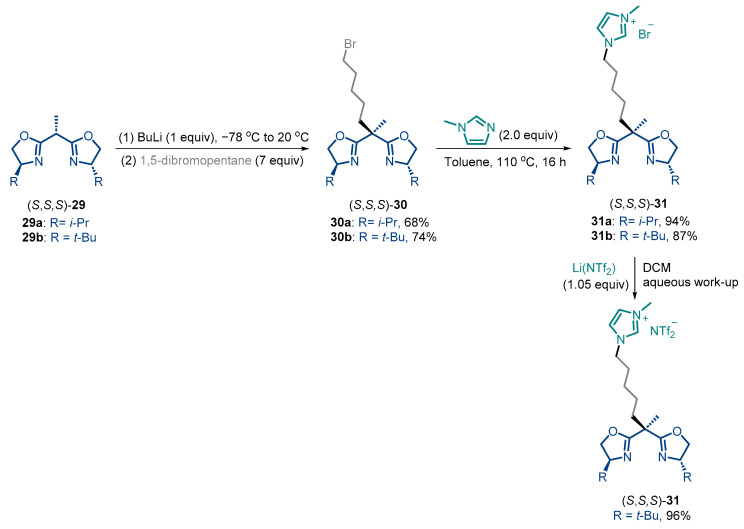
Synthesis of imidazolium-salt-tagged bisoxazoline ligands.

The synthesis of imidazolium-salt-containing bisoxazoline ligands is outlined in Scheme 7. The readily available oxazolines (*S*,*S*,*S*)-**29** were prepared from commercially available diethyl methylmalonate and (*S*)-valinol or (*S*)-*tert*-leucinol, followed by cyclization with DAST and deprotonation with stoichiometric BuLi. The deprotonated intermediates were then treated with excess 1,5-dibromopentane, yielding the desired bromopentyl-substituted bisoxazolines (*S*,*S*,*S*)-**30**. The terminal bromide was reacted with 1-methylimidazole in refluxed toluene to afford the corresponding bromide salts (*S*,*S*,*S*)-**31** in good yields. Notably, the bromide anion in (*S*,*S*,*S*)-**31b** was successfully exchanged for NTf_2_ by vigorous stirring in the LiNTf_2_ aqueous solution, providing (*S*,*S*,*S*)-**31c** in a 96% yield.

**Scheme 8:**
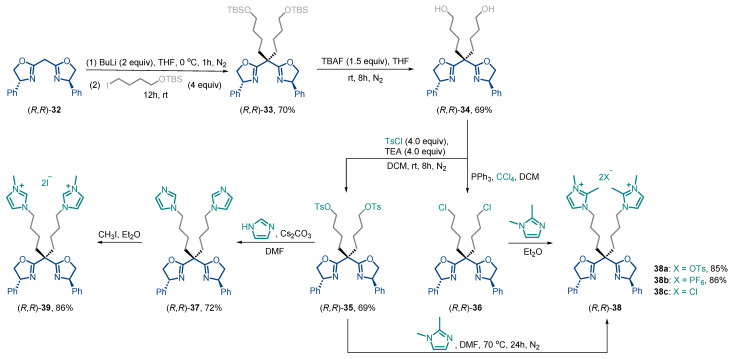
Select example on synthesis of *C*_2_-symmetric ionic-liquid-supported bisoxazolines.

Later in 2010, Zhou presented an enhanced system for catalyst robustness using imidazolium-salt-loaded *C*_2_-symmetric bisoxazolines and successfully completed 20 catalytic cycles of Cu-catalyzed asymmetric Diels–Alder reactions in ionic liquids [58]. The ionic-liquid-soluble *C*_2_-symmetric oxazolines were prepared via two different routes, as shown in Scheme 8. The hydroxyl-substituted bisoxazoline (*R*,*R*)-**34** was first converted into a tosyl-substituted bisoxazoline (*R*,*R*)-**35** using TsCl and then chlorinated to form bisoxazoline (*R*,*R*)-**36** via the Appel reaction. The resulting functionalized oxazolines were then reacted with 1,2-dimethylimidazole in DMF to yield the imidazolium-labeled ligands (*R*,*R*)-**38**. Using this synthetic route, ionic-liquid-supported bisoxazolines containing various chiral side chains (**40**-**41**) and carbon linkers of differing lengths ((*S*,*S*)-**42**) were successfully prepared. The specific structures of these ligands are provided in Table 4.

Alternatively, tosyl-substituted bisoxazoline (*R*,*R*)-**35** was derived from imidazole-substituted oxazolines (*R*,*R*)-**37** by reaction with imidazole in DMF, catalyzed by Cs_2_CO_3_. The subsequent quaternization of the imidazole-substituted bisoxazoline with CH_3_I yielded the target iodide salt (*R*,*R*)-**39**.

### 3.3. Diphenylethylenediamine (DPEN) Ligands

Over the past three decades, chiral ligands derived from the diphenylethylenediamine (DPEN) framework have demonstrated remarkable success in transition-metal catalysis, particularly in the enantioselective reduction of prochiral ketones and imines [59,60,61,62]. In 2012, Deng reported a class of chiral amphiphilic surfactant ligands with hydrophobic alkyl chains and hydrophilic ionic groups, based on TsDPEN ligands as the core structure [63]. In aqueous systems, the produced ligands and corresponding catalysts formed micelles through self-assembly, as confirmed by TEM analyses, and successfully catalyzed asymmetric transfer hydrogenation (ATH) of aliphatic ketones.

**Scheme 9:**
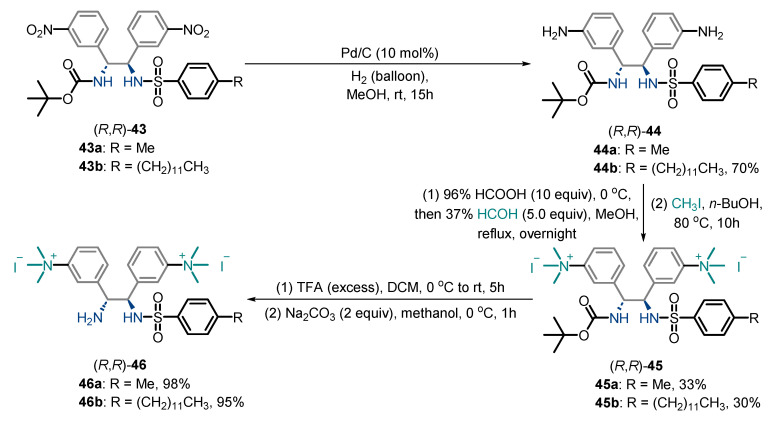
Synthetic route of chiral surfactant-type DPEN ligands.

As shown in Scheme 9 from Deng’s work, the nitro group in the starting material (*R*,*R*)-**43** was reduced to an amino group, and the hydrophobic alkyl chain was introduced, resulting in the key intermediates (*R*,*R*)-**44**. The intermediates were then dissolved in methanol and reacted with formic acid at 0 °C, followed by careful addition of a 37% aqueous formaldehyde solution. The resulting mixture was refluxed overnight. The crude products were then directly quaternized with CH_3_I, yielding iodide salts (*R*,*R*)-**45** in an over 30% yield. The protecting groups on the amines were removed prior to ligand utilization by reacting with excess TFA at room temperature.

Later, Kawasaki and Nishide employed different strategies to synthesize TsDPEN derivatives with various ionic molecules [64]. They investigated the effect of the structure, particularly the quaternary ammonium group and the linker alkyl chain, on catalytic ability and recyclability in the ATH of acetophenone with ILs. The enantioselectivity of the obtained products was found to be highly dependent on the structure of the ionic moiety and the length of the alkyl chain connecting the ligand site and the ionic group, as evidenced by the catalytic results.

**Scheme 10:**
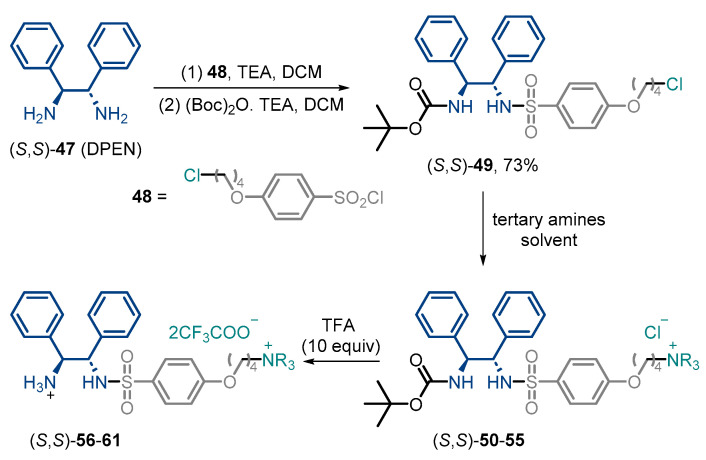
Synthetic route of chiral ionic-liquid-supported TsDPEN derivatives.

In Kawasaki and Nishide’s work, the quaternary ammonium fragments in the recyclable chiral TsDPEN ligands were incorporated into the toluene sulfonyl unit, employing a completely different synthetic strategy compared to the ligands designed by Deng, where the ionic liquid moiety was loaded onto the benzene ring of the DPEN skeleton. Therefore, the TsDPEN derivative with an alkyl chloride (*S*,*S*)-**49** was chosen as the starting material for preparing the final ligands (Scheme 10). The free chloride of (*S*,*S*)-**49** was reacted with a variety of amines, resulting in the quaternary ammonium compounds (**50**–**55**) with moderate to high yields, as shown in Table 5. The subsequent reaction with trifluoroacetic acid cleaved the protecting *tert*-butoxycarbonyl group and completed the anion exchange process, yielding the final ionic-liquid-supported TsDPEN ligands (**56**–**61**, Table 6).

Recently, Bica reported the incorporation of water-soluble pyridinium salt moieties into DPEN ligands, successfully achieving the ATH of aliphatic and aromatic ketones, as well as imine substrates, using ruthenium complexes under aqueous phase conditions [65]. The introduction of pyridinium salt fragments, as studied by DFT calculations, revealed that the cationic molecules have little effect on the electronic properties of the chiral *N*,*N*-skeleton.

**Scheme 11:**
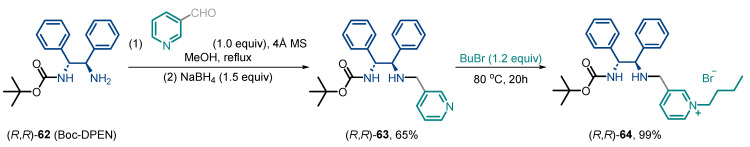
Synthesis of water-soluble pyridinium-tagged DPEN ligands.

The synthetic route for the water-soluble DPEN-type ligands presented by Bica’s group is depicted in Scheme 11. Taking the synthesis of a typical ligand (*R*,*R*)-**64** as an example, Boc-protected DPEN (*R*,*R*)-**62** was dissolved in anhydrous methanol and reacted with freshly distilled pyridine-3-carbaldehyde. The crude product was then directly subjected to reduction of the imine group with NaBH_4_ to yield intermediate (*R*,*R*)-**63**, which contained the desired pyridine unit. This intermediate was subsequently reacted with bromoalkanes of varying chain lengths to introduce the desired ionic groups, achieving nearly quantitative yields. The structures and corresponding preparation yields for the quaternization step of the pyridinium-tagged ligands (**65**–**70**) synthesized in Bica’s report are shown in Table 7.

### 3.4. Salen-Type Ligands

Since their independent development by Jacobsen and Katsuki in the 1990s, *N*,*N*’-bis(salicylidine)-ethylenediamine (Salen) derivatives have been recognized as some of the most successful ligands for electrophilic addition reactions, particularly in the activation of epoxides and carbonyl compounds [66,67,68,69]. In 2016, Jing’s group described the synthesis of Salen-based ligands containing imidazolium salt fragments, as well as their corresponding Co-complexes [70]. The researchers successfully employed these catalysts to achieve the asymmetric cycloaddition of CO_2_ with propylene oxide, yielding chiral epoxides in high yields and moderate enantioselectivity.

**Scheme 12:**
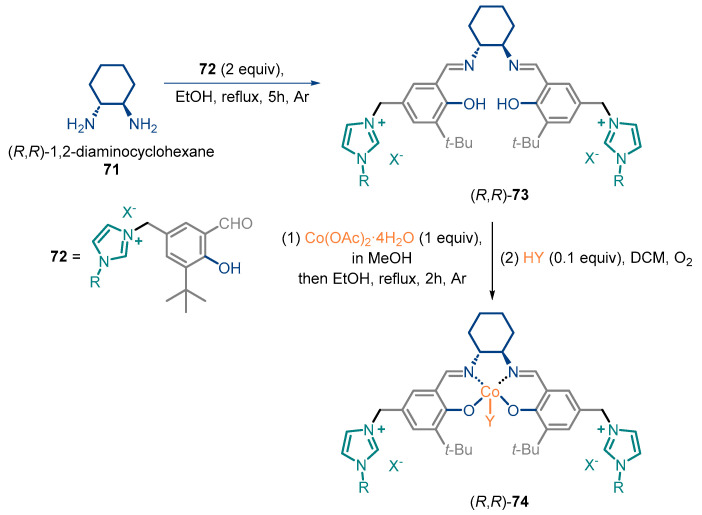
General synthetic procedure of imidazolium-tagged Salen ligands and corresponding catalysts.

The synthesis of recyclable ligands and their corresponding catalysts, as developed by Jing’s group, is illustrated in Scheme 12. 1,2-Diaminocyclohexane (*R*,*R*)-**71** and N-alkyl imidazolium salicylaldehydes **72** were dissolved in ethanol, and the resulting solution was refluxed for five hours under an inert atmosphere. Afterward, the solvent was evaporated to near dryness, and the solution was diluted with hexane to precipitate the crude product (*R*,*R*)-**73**, which was washed with hexane and dried under vacuum.

Subsequently, the transition-metal precursor (Co(OAc)_2_·4H₂O, selected for this reaction) was added to an ethanol solution of the prepared *N*-imidazolium-tagged Salen ligands (*R*,*R*)-**73**. The resulting solution was refluxed for two hours, leading to the formation of the crude Salen-Co(II) complex. The desired catalysts, the Salen-Co(III)Y complex (*R*,*R*)-**74**, were obtained by oxidizing the Salen-Co(II) complex in dichloromethane, in the presence of the relevant acid, under an oxygen atmosphere. The selected structures of the obtained Salen-Co(III)Y complexes (*R*,*R*)-**74** are listed in Table 8.

### 3.5. Chiral Ligands with 1,1′-Binaphthene Skeleton

In 2013, Jin’s group reported the first case of BINAP-type ligands containing imidazolium moieties and their corresponding Ru-catalyzed asymmetric hydrogenation of *β*-keto esters, achieving high stereoselectivity with the prepared ligands [71]. Notably, the use of BINAP-type imidazolium salts in an ionic liquid system exhibited a comparable degree of stereoselectivity in the reduction products to those obtained using conventional unsupported (*S*)-BINAP parent ligands. The incorporation of ionic liquids in the catalyst recovery process was shown to be an effective strategy for minimizing catalyst loss and slowing catalyst oxidation.

**Scheme 13:**
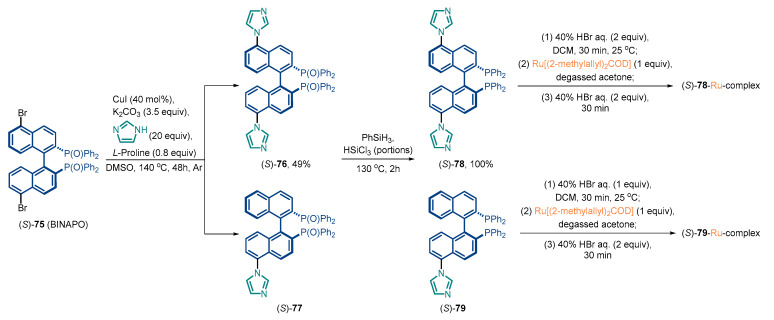
Synthesis of 5,5′-diimidazolium-loaded BINAP ligands and corresponding Ru-complexes.

The synthetic routes for BINAP ligands containing imidazolium moieties are illustrated in Scheme 13. The target ligands were strategically designed with charge-containing groups positioned at the 5,5′-substitution sites. Optically active (*S*)-5,5′-dibromo BINAP dioxide ((*S*)-**75**, BINAPO) was selected as the most suitable starting material, and the imidazole group was introduced at the designed position of BINAPO via an *L*-proline-promoted Cu-catalyzed C-N bond formation. The subsequent reduction of phosphine oxide (*S*)-**76** using a mixture of PhSiH_3_ and HSiCl_3_ quantitatively yielded 5,5′-diimidazole BINAP (*S*)-**78**. The final HBr salt was prepared in situ using a 40% aqueous HBr solution. Additionally, 5-imidazole BINAP (*S*)-**79**, prepared as a by-product during the above process, was also evaluated as a potential ligand for the enantioselective hydrogenation of *β*-keto esters.

**Figure 7:**
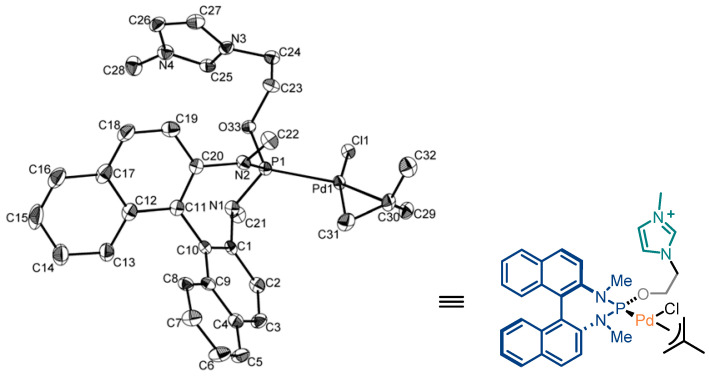
The molecular view of Pd-imidazolium-salt-supported phosphate complex (cation). Reprinted with permission from Ref. [72]. Copyright 2014 American Chemical Society.

Subsequently, Gómez and Rocamora reported the development of novel imidazolium-salt-supported chiral phosphate ligands with a 1,1′-binaphthene backbone and conducted a structural characterization of their Pd complexes (Figure 7) [72]. The ionic-liquid-supported catalysts were then applied in asymmetric allylic alkylation, amination, and sulfonylation using *rac*-3-acetoxy-1,3-diphenyl-1-propene as a substrate in neat ionic liquid systems. The most effective asymmetric induction was achieved by utilizing the preformed complex as a catalyst, resulting in 76% *ee* for the amination and 72% *ee* for the sulfonylation, with conversions exceeding 60%. Furthermore, the recycling procedure of the Pd-complex in asymmetric allylic amination in [Pyr][NTf_2_] was also investigated in Gómez and Rocamora’s study.

**Scheme 14:**
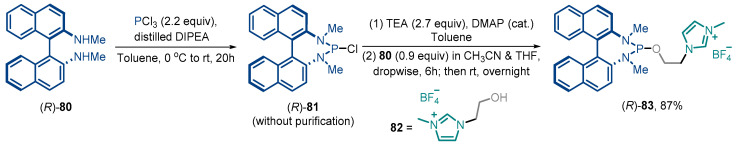
Synthesis of chiral imidazolium-salt-supported phosphate ligands.

The preparation process for the novel ligand with a 1,1′-binaphthene skeleton is shown in Scheme 14. The starting material, (*R*)-*N*,*N*’-dimethyl-1,1′-binaphthyl-2,2′-diamine (*R*)-**80**, was dissolved in toluene along with freshly distilled *i*-Pr_2_EtN (DIPEA). The mixture was then treated with a PCl_3_ solution in toluene at 0 °C. The reaction was stirred for 20 h at room temperature to facilitate the formation of the crucial diamidophosphite intermediate (*R*)-**81**. This intermediate was then directly treated with a solution of 1-(2-hydroxyethyl)-3-methylimidazolium tetrafluoroborate **82** in a combined solvent of acetonitrile and THF over a period of six hours, in the presence of NEt_3_ and a catalytic amount of DMAP. After the reaction was complete, the yellow-colored target product (*R*)-**83** was obtained without further purification.

### 3.6. N,O-Ligands (Metallomicellar Catalysts)

In 2015, Bica introduced a methodology for synthesizing and applying chiral ionic liquids [73]. This research utilized natural amino alcohols—prominent chiral ligands—for structural modifications. By incorporating pyridinium fragments into the amino alcohol backbone, subsequent quaternization reactions yielded a series of water-soluble pyridinium-salt-functionalized chiral ionic liquids. Catalytic results of asymmetric transfer hydrogenation conducted in aqueous phases demonstrated that the incorporation of water-soluble fragments in the ligands enhanced the catalytic efficiency and recoverability of the catalysts, compared to the neutral parent structure.

**Scheme 15:**
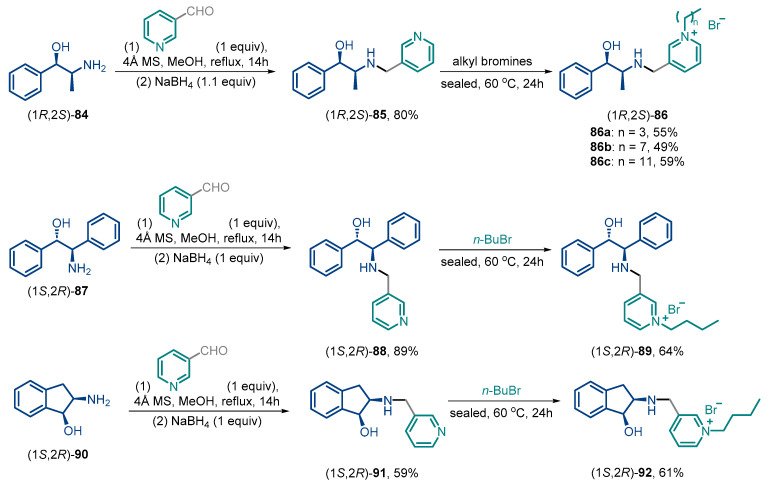
Synthesis of chiral pyridinium-tagged water-soluble ionic liquids.

The strategy for synthesizing these water-soluble chiral ionic liquids is shown in Scheme 15. The compound (1*R*,2*S*)-**86a** is taken as an example. The amino group in the starting material (1*R*,2*S*)-norephedrine **84** was refluxed with freshly distilled pyridine-3-carbaldehyde in anhydrous methanol, using activated molecular sieves as dehydrating agents, to give the corresponding imine intermediates. The intermediate products were then directly reduced using sodium borohydride at room temperature, resulting in the formation of pyridine-fragment-loaded compounds (1*R*,2*S*)-**85** with a high yield. Next, the purified (1*R*,2*S*)-**85** was stirred with an n-butyl bromide mixture at 60 °C for 24 h to form the final ionic liquids (1*R*,2*S*)-**86a**. It is noteworthy that the pyridinium-salt-supported chiral ionic liquid compounds were successfully purified by preparative HPLC. Other pyridinium-salt-supported chiral ionic liquids (**89** and **92**) designed by Bica’s group are also shown in Scheme 15.

Shi and Wang, in 2020, reported a series of Schiff base ligands for asymmetric Michael addition in water [74]. They confirmed the formation of metallomicellar structures under aqueous conditions for the corresponding Lewis acid complexes using scanning electron microscopy (SEM). As a result, further asymmetric Michael additions occurred within the interior of these metallomicelles.

**Scheme 16:**
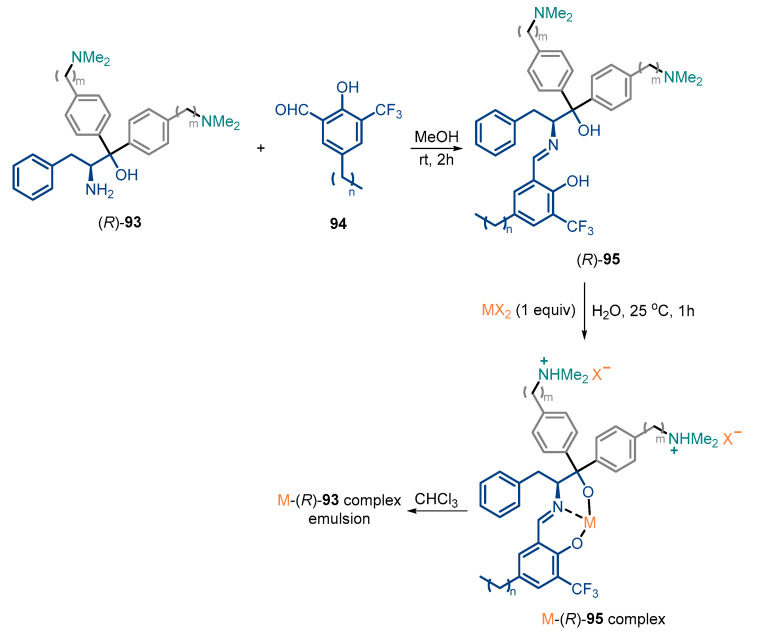
Synthesis of chiral Schiff base ligands reported by Shi and Wang.

The research group also presented the synthetic route for the Schiff base ligands and corresponding transition-metal catalysts, as illustrated in Scheme 16. A solution of the selected chiral amino alcohol (*R*)-**93** in methanol was treated with the readily prepared salicylaldehyde derivatives **94**. This solution was agitated for two hours at room temperature. The solvent was then removed under reduced pressure, and the residue was purified by column chromatography using DCM/MeOH as the eluent. Prior to initiating the catalytic reaction within an aqueous phase system, the purified Schiff base ligands (*R*)-**95** were treated with various metal precursors, including Zn(OTf)_2_, Cu(OTf)_2_, and Sc(OTf)_3_. This treatment resulted in the formation of the desired quaternary-ammonium-tagged M-(*R*)-**95** complexes, which existed in the form of metallomicelles in an organic solvent/water combined system. The structures of the prepared Schiff base ligands (*R*)-**95** by Shi and Wang are presented in Table 9.

### 3.7. Imidazolidinone Ligand

In 2003, Lee reported the application of a novel imidazolium-salt-tagged chiral bisphosphine ligand and its corresponding Rh complex with an imidazolidin-2-one backbone for the asymmetric hydrogenation of N-acetylphenylethenamine [75]. The imidazolium-salt-tagged catalysts demonstrated enhanced recoverability compared to the unsupported bisphosphine-derived catalysts with the same backbone, as indicated in Lee’s work. Additionally, testing results of the Rh content in the *i*-PrOH layer by ICP-OES showed that the attachment of an imidazolium ionic tag significantly increased the solubility of the catalysts in ILs and reduced catalyst leaching during the recycling process.

**Scheme 17:**
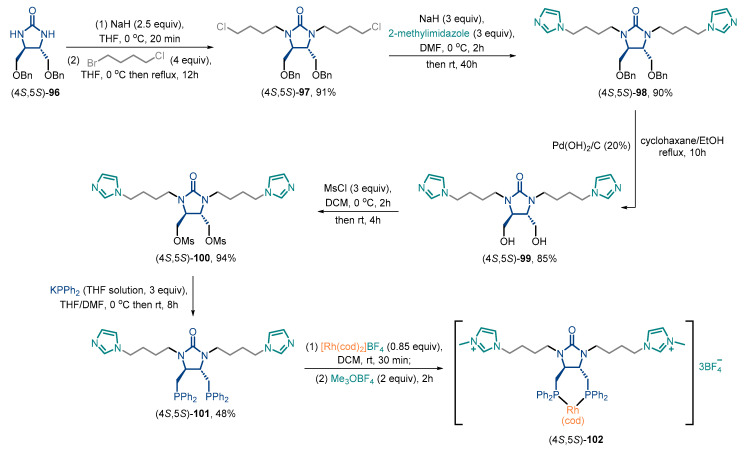
Synthesis of chiral imidazolium-salt-tagged bisphosphine ligand and corresponding Rh-complex.

The skeletal structure of the ligand was derived from the selected raw material, (4*S*,5*S*)-bis(benzyloxymethyl)imidazolidin-2-one **96** (Scheme 17). This compound was reacted with 1-bromo-4-chlorobutane in the presence of a sodium hydride suspension, yielding (4*S*,5*S*)-**97**. The terminal chlorine of the alkyl chain in (4*S*,5*S*)-**97** was subsequently replaced with an imidazole group through a reaction with 2-methylimidazole, resulting in the key intermediate (4*S*,5*S*)-**98**. Lee then outlined a three-step sequential transformation process for introducing the phosphine moiety into the backbone structure of the chiral ligand, culminating in (4*S*,5*S*)-101. The resulting bisphosphine compound (4*S*,5*S*)-**101** was treated with [Rh(cod)_2_]BF_4_ in DCM, followed by N-methylation with trimethyloxonium tetrafluoroborate to yield the Rh complex (4*S*,5*S*)-**102**.

## 4. Transition-Metal Precursors Used in Catalyzed Stereoselective Transformations Within Ionic-Liquid-Supported Ligands

In the preceding section, the synthetic schemes for chiral ionic-liquid-supported ligands were presented clearly using reaction formulas. The synthesis of transition-metal complexes with functionalized ligands is also incorporated into the synthesis routes within the specific schemes.

In particular, the choice of transition-metal precursors, which is crucial for the selective outcome of asymmetric catalytic reactions, have been the subject of our research. It is evident that a variety of transition metals have been employed in asymmetric reactions, each with distinct catalytic mechanisms. Furthermore, in asymmetric transformations that share the same mechanism, the metal precursors are often distinguished by structural differences in the ligands used. In this section, we provide an overview of transition-metal precursors that have yielded promising results when paired with the aforementioned chiral ligands. This overview, presented in Table 10, aims to assist scholars and readers of this review in quickly grasping the scope of the transition-metal precursors involved.

## 5. Select Experimental Procedures on Synthesis of Chiral Ionic-Liquid-Supported Ligands and Catalysts

In this review, we aim to present the structures of the reported chiral ionic-liquid-supported ligands and provide an overview of the synthesis processes for the representative ligands discussed above. The synthetic schemes outlined in the previous section are concise, enabling scholars and readers to quickly grasp the key steps involved in the ligand synthesis. However, it is apparent that the procedures influencing the yield and purity of the target ionic-liquid-supported products, as well as key factors such as reagent selection, catalysts, and reaction conditions, have not been fully elaborated. Therefore, we seek to offer a comprehensive account of the critical schemes associated with the synthetic routes discussed. This will help raise awareness of potential issues in the synthesis of chiral ionic-liquid-supported ligands, aiding scholars in the efficient development of comprehensive synthetic pathways and in the design of novel chiral ionic-liquid-supported ligands and catalysts.

It is important to note that ethical considerations prevent the direct replication of experimental procedures from the original literature without appropriate modifications. Therefore, in the following description, we will make every effort to accurately reproduce the original synthetic approaches. Notably, the detailed characterization of the reported compounds has been intentionally omitted to allow scholars and readers to focus on the procedural aspects of their preparation methods. Reference numbers are provided alongside the synthetic steps, granting access to the original synthesis protocols and supplementary analytical data related to most of these compounds.

### 5.1. Construction on Backbone of Chiral Ionic-Liquid-Supported Ligands

In certain references, the authors have provided a comprehensive synthetic route to the chiral ligand, together with the original manuscript and the associated supplementary material. It is important to consider the target ionic-liquid-supported ligands at the outset of their design and synthesis process, with a view to facilitating the subsequent introduction of the ionic liquid fragment. Consequently, the ligands under discussion in short review were distinct from those of unsupported parent ligands in terms of starting materials, reaction reagents, and transformation conditions. In this part, we attempted to demonstrate three typical ligands with different backbone structures, focusing on the strategies employed in the construction of the basic catalytic active structures, particularly the introduction of chelating groups.

**Example 1.** Synthesis of (*R*,*Rp*)-4-hydroxymethyl-2-[(2-diphenylphosphino)ferrocenyl] oxazoline (**26**)

**Scheme 18:**
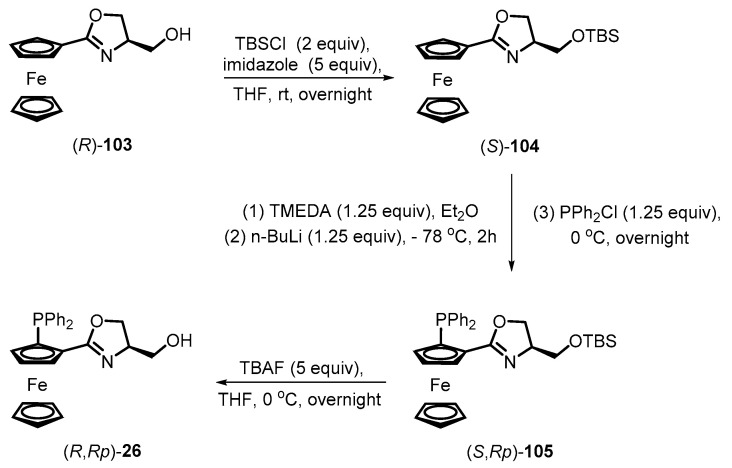
Synthesis of (*R*,*Rp*)-4-hydroxymethyl-2-[(2-diphenylphosphino)ferrocenyl] oxazoline (**26**).

The preparation approach for (*R*,*Rp*)-**26** was presented in Scheme 18. A solution of (*R*)-4-hydroxymethyl-2-ferrocenyloxazoline (**103**, 1.0 g, 3.5 mmol) in THF (20 mL) was prepared. Subsequently, imidazole (1.07 g, 15.8 mmol, 5 equiv) was added, followed by the addition of TBSCl (1.05 g, 7 mmol, 2 equiv) at room temperature. The reaction was then stirred for a period of 24 h and subsequently quenched with brine. The organic layer was subsequently separated, dried over MgSO_4_, and concentrated under reduced pressure. The crude product was purified by column chromatography on silica gel (eluent: petroleum ether/EtOAc = 15:1) to afford (*S*)-4-(*tert*-butyldimethylsilyloxy)methyl-2-ferrocenyloxazoline (**104**, 1.37 g, 98%) as a dark red liquid.

In an inert atmosphere, a solution of (*S*)-**104** (1.615 g, 4.04 mmol) in dry ether (50 mL) was degassed and then treated with TMEDA (610 mg, 5.25 mmol, 1.25 equiv) at room temperature. The resulting mixture was cooled to -78 °C, and *n*-BuLi (3.3 mL, 1.6 M, 5.28 mmol, 1.25 equiv) was then added dropwise and stirred at this temperature for 2 h. Subsequently, the mixture was treated with PPh_2_Cl (1.16 g, 5.25 mmol, 1.25 equiv) and stirred for 12 h at 0 °C. Subsequently, the reaction mixture was quenched with a saturated aqueous solution of NaHCO_3_ and diluted with ether. The organic layer was extracted with ether, and the combined organic phase was dried over MgSO_4_ and concentrated in vacuo. The crude product was purified by column chromatography on silica gel (eluent: petroleum ether/EtOAc = 10:1) to yield (*S*,*Rp*)-4-(*tert*-butyldimethylsilyloxy)methyl-2-[(2-diphenylphosphino)ferrocenyl] oxazoline (**105**, 2.10 g, 89%) as a yellow solid.

A degassed solution of (*S*,*Rp*)-**105** (550 mg, 0.94 mmol) in dry THF (20 mL) was treated with tetrabutylammonium fluoride (TBAF) (1.24 g, 4.75 mmol, 5 equiv) at 0 °C. The resulting reaction mixture was stirred overnight and quenched with water. The organic layer was extracted with ether on three times. The combined organic layer was dried over MgSO_4_ and concentrated in vacuo. The crude product was purified by column chromatography on silica gel (eluent: petroleum ether/EtOAc/EtOH = 10:10:1) to provide (*R*,*Rp*)-**26** (379 mg, 86%) as an orange solid.

Characterization of the above prepared oxazolinyl ferrocenes in Scheme 18, see ref. [54].

**Example 2.** Synthesis of (*S*)-5,5′-diimidazole BINAP (**78**) and (*S*)-5-imidazole BINAP (**79**)

The preparation route has been shown in Scheme 13. The mixture comprised (*S*)-**75** (3.6 g, 4.4 mmol), imidazole (6.1 g, 89.0 mmol, 20 equiv), K_2_CO_3_ (2.5 g, 18.0 mmol, 3.5 equiv), CuI (0.35 g, 1.8 mmol, 0.4 equiv), and *L*-proline (0.42 g, 3.6 mmol, 0.8 equiv) in a solution of 70 mL of DMSO, which was stirred at 140 °C for 48 h under an argon atmosphere. Once cooled, the mixture was filtered, and the filtrate was then treated with ethyl acetate. The organic layer was subjected to a washing process with brine, followed by drying using Na_2_SO_4_ and subsequent concentration under vacuum. Purification of the residual solid by silica gel column chromatography (eluent: EtOAc/EtOH/H_2_O = 15:2:1) resulted in the isolation of pure (*S*)-**76** as a white solid (yield 1.7 g, 49.1%). Meanwhile, 5-imidazole BINAPO (*S*)-**77** was obtained as a byproduct (yield 0.78 g) through the Cu-catalyzed C-N bond formation process.

A solution comprising a mixture of (*S*)-**76** (0.2 g, 0.26 mmol) and phenylsilane (PhSiH_3_) (1.5 mL, 12.2 mmol, 10 equiv) was subjected to a heating process at 130 °C. Three portions of trichlorosilane (HSiCl_3_) (0.4 mL) were introduced into the reaction mixture by syringe after 1 h, 3 h, and 15 h, respectively, and the reaction was stirred for another 2 h. Following this, the mixture was cooled, and the volatiles were removed under reduced pressure. The residue solid was then washed with degassed cyclohexane, filtered, and evaporated, and the desired product (*S*)-**78** was obtained in a quantitative yield.

Notably, the 5-imidazole BINAP (*S*)-**79** was prepared using the same procedure for (*S*)-**78**. For further analytical data on related 1,1′-binaphthene skeleton ligands, see ref. [71].

**Example 3.** Synthesis of (4*S*,5*S*)-4,5-bis(diphenylphosphinomethyl)-*N*,*N’*-bis [4-(2-methylimidazol-1-yl)butyl] imidazolidin-2-one (**101**)

The brief preparation route has been presented in Scheme 17. The detailed scheme was started from (4*S*,5*S*)-4,5-bis(benzyloxymethyl)-*N*,*N’*-bis [4-(2-methylimidazol-1-yl)butyl]imidazolidin-2-one (**98**). A solution of (4*S*,5*S*)-**98** (1.1 g, 2.36 mmol) in cyclohexane was prepared and then was conducted by refluxing a solvent comprising cyclohexene (14 mL) and ethanol (20 mL), in the presence of a catalyst 20% Pd(OH)_2_/C (0.5 g), for a period of 10 h. Upon cooling to room temperature, the catalyst was filtered through celite, and the solution was concentrated under reduced pressure. The residue was further purified by column chromatography on silica gel (eluent: DCM/MeOH = 5:1) to yield (4*S*,5*S*)-4,5-bis(hydroxymethyl)-*N*,*N’*-bis[4-(2-methylimidazol-1-yl)butyl]imidazolidin-2-one (**99**) as a pale yellowish oil (0.65 g, 85%).

The stirring solution comprised (4*S*,5*S*)-**99** (0.4 g, 0.96 mmol) and TEA (0.80 mL, 5.73 mmol, 6 equiv) in DCM (20 mL). At 0 °C, methane sulfonyl chloride (0.22 mL, 2.87 mmol, 3 equiv) was added to this mixture. The reaction was agitated at room temperature for 4 h. The reaction was terminated through the addition of water at 0 °C, and the aqueous layer was extracted with DCM. The combined organic layer was dried over MgSO_4_, filtered, and the filtrate was concentrated by evaporation. The crude residue was purified by column chromatography on silica gel (eluent: DCM/MeOH = 5:1) to afford (4*S*,5*S*)-4,5-bis(hydroxymethyl)-*N*,*N*’-bis[4-(2-methylimidazol-1-yl)butyl]imidazolidin-2-one (**100**, 0.48 g, 94%) as a colorless oil.

The aforementioned (4*S*,5*S*)-**100** (30 mg, 0.06 mmol) was dissolved in a solution of THF and DMF (volume ratio 10:1). Potassium diphenylphosphide (0.36 mL of a 0.5 M solution in THF, 0.18 mmol, 3 equiv) was added at 0 °C, and the reaction mixture was stirred at room temperature for 8 h. The precipitate was filtered off through celite and the residue was washed with benzene. The filtrate was subsequently evaporated, after which the residue was purified via column chromatography on silica gel (eluent: DCM/MeOH = 6:1) to furnish the desired bisphosphine (4*S*,5*S*)-**101** (20 mg, 48%). For characterization of the prepared imidazolidin-2-one compounds in Lee’s work, see ref. [75] and corresponding supporting information.

### 5.2. Introduction Processes of Linker in the Supported Ligands

The principal objective of *linker* is to establish an effective connection between the backbone of a chiral ligand and a suitable ionic liquid fragment. The variety of types of linking groups was extensive, and their selection was typically based on the structural units available in the chiral ligands, as well as the specific structure of the ionic liquid fragments. Consequently, the methods for introducing different types of *linker* groups exhibited considerable diversity. The objective of this part was to provide an overview of the synthesis of common *linker* in the structures of chiral ionic-liquid-supported ligands, including amides (including sulfonamides), imines, phosphite groups, and alkyl chains.

#### 5.2.1. Amide and Sulfonamide Groups as the *Linker*

**Example 4.** Synthesis of (*R*,*Sp*)-**11**

The solution of *N*,*N*’-dicyclohexylcarbodiimide (DCC) (128 mg, 0.62 mmol, 2 equiv) in THF (2 mL) was introduced gradually to the solution of (*R*,*Sp*)-**10a** (200 mg, 0.31 mmol) and 3-(3-aminopropyl)-1-methylimidazolium bis(trifluoromethylsulfonyl)amide (134 mg, 0.37 mmol, 1.2 equiv) in THF (8 mL) at 0 °C under argon. The reaction mixture was then allowed to reach room temperature and stirred for a further 24 h until no starting material was detected on thin layer chromatography. Following the removal of the organic solvent under vacuum, the residual solids were re-dissolved in 10 mL of chloroform. The white solids were filtered and the organic solvent was removed, and the remaining material was subjected to chromatography on silica gel (eluent: DCM/MeOH = 10:1). The desired product (*R*,*Sp*)-**11a** was isolated as an orange solid, with a yield of 200.2 mg (0.19 mmol, 72%).

The similar imizolium-tagged Josiphos ligands (*R*,*Sp*)-**11b** was prepared with the same procedure described for (*R*,*Sp*)-**11a** with a yield of 75% after purification of column chromatography on silica gel (eluent: DCM/MeOH = 15:1). For characterization of the prepared Josiphos ligands, see ref. [49].

**Example 5**. Synthesis of (*R*,*Sp*)-6-bromo-*N*-[1-[2-(diphenylphosphanyl)ferrocen-1-yl]ethyl]-*N*-methyl hexanamide (**2a**)

The amine (*R*,*Sp*)-**1a** (927 mg, 2.33 mmol) and TEA (0.49 mL, 354 mg, 3.50 mmol, 1.5 equiv) were dissolved in anhydrous diethyl ether (30 mL) to create a solution. Subsequently, Br(CH_2_)_5_COCl (747 mg, 3.50 mmol, 1.5 equiv) was added dropwise to the solution using a syringe. The resulting mixture was allowed to stand at room temperature for 1 h, during which time a white precipitate was formed. Aqueous NaOH (1 M, 25 mL) was then added, and the phases were separated. The aqueous phase was extracted with diethyl ether. The combined organic phase was then dried using anhydrous Na_2_SO_4_ and concentrated. The pure compound (*R*,*Sp*)-**2a** (986 mg, 70%) was isolated as an orange oil after an isolation by column chromatography on silica gel (eluent: hexane/EtOAc = 9:1).

(*R*,*Sp*)-6-Bromo-*N*-[1-[2-(phenylselanyl)ferrocen-1-yl]ethyl]-*N*-methylhexanamide **2b** was synthesized by the indicated process and gave a yield of 88% after purification by column chromatography on silica gel (eluent: hexane/EtOAc = 9:1) as an orange oil. For characterization of the prepared ferrocene amides, see ref. [46].

**Example 6.** Synthesis of (*S*,*S*)-*N*-[2-[(1,1-dimethylethoxy)carbonyl]amino-1,2-diphenylethyl]-4-(4-chlorobutoxy)benzene sulfonamide (**49**)

(1*S*,2*S*)-1,2-DPEN (**47**, 498 mg, 2.34 mmol) and TEA (0.340 mL, 2.44 mmol, 1.05 equiv) were added to a solution of 4-(4-chlorobutoxy)benzene sulfonyl chloride (**48**, 346 mg, 1.22 mmol, 0.5 equiv) in DCM (3 mL) at 0 °C under a nitrogen atmosphere. Following a 22-h period of stirring at room temperature, a solution of (Boc)_2_O (400 mg, 1.83 mmol, 0.8 equiv) and TEA (0.255 mL, 1.83 mmol, 0.8 equiv) in DCM (2 mL) was added to the solution under nitrogen. The mixture was stirred for a further 24 h at room temperature. After the completion of the reaction, the DCM solvent was removed before the saturated NaHCO_3_ (5.0 mL) was added to the residue. The crude product was extracted with 20 mL of EtOAc, and the organic layer was subjected to drying with Na_2_SO_4_ and was purified by column chromatography (eluent: hexane/EtOAc = 3:1) to afford pure (*S*,*S*)-**49** with an overall yield of 498 mg (73%). For characterization of (1*S*,2*S*)-**49**, see ref. [64].

#### 5.2.2. Imines as the *Linker*

**Example 7.** General synthesis of (*R*,*Sp*)-**14**

(*R*,*Sp*)-PPFNH_2_ (**12**, 433 mg, 1.05 mmol, 1.05 equiv), 4-formyl-*N*,*N*,*N*-trimethyl-benzenaminium iodide (**13a**, 291 mg, 1 mmol), and MgSO_4_ (500 mg) were conducted in an argon-filled Schlenk tube with absolute alcohol as the solvent. The reaction was allowed to proceed at reflux temperature for 4 h, after which the MgSO_4_ was removed by filtration. Following the removal of the solvent under vacuum, the crude was purified by washing with ethyl ether. After recrystallisation from DCM, the final ligand (*R*,*Sp*)-**14a** was gained as a yellow solid with 93% yield (638 mg). A detailed characterization of the prepared quaternary ammonium-salt-tagged ferrocenes (*R*,*Sp*)-**14** can be found in ref. [50].

**Example 8.** General synthesis of ligands (*R*,*R*)-**73**

(1*R*,2*R*)-1,2-Diaminocyclohexane (**71**, 0.5 mmol) and the readily prepared *N*-alkylimidazolium salicylaldehyde (**72**, 1.0 mmol, 2 equiv) were dissolved in ethanol (10.0 mL) in a two-necked round-bottomed flask equipped with a condenser and a gas protector. The combined solution was heated under reflux for a period of 5 h, with an argon atmosphere maintained. The solvents were then evaporated to near-dryness and a solution of hexane (15 mL) was used for precipitation of the crude product (*R*,*R*)-**73**, which was filtered, washed with hexane, and subsequently dried under a vacuum. For characterization of the prepared Salen-type ligands (*R*,*R*)-**73** prepared by the same method, see ref. [70].

#### 5.2.3. Phosphite Group as the *Linker*

**Example 9.** Synthesis of (*R*)-**83**

The synthetic routes of (*R*)-**83** have been described in Scheme 14. (*R*)-*N*,*N*′-dimethyl-1,1′-binaphthyl-2,2′-diamine (**80**, 0.5 g, 1.6 mmol) was dissolved in 10 mL of toluene with the freshly distilled and dried DIPEA. A solution of PCl_3_ (0.30 mL, 3.5 mmol, 2.2 equiv) in 5 mL of toluene was added dropwise at 0 °C. Following a stirring period of 20 h at ambient temperature, the solution was concentrated by evaporation.

The resulting solid (*R*)-**81**, dissolved in 10 mL of toluene, was then treated with a catalytic amount of DMAP (2.6 mg, 0.021 mmol) and TEA (0.60 mL, 4.3 mmol, 2.7 equiv). Subsequently, a solution of 1-(2-hydroxyethyl)-3-methylimidazolium tetrafluoroborate (**82**, 0.30 g, 1.4 mmol, 0.9 equiv) in acetonitrile (2 mL) and THF (4 mL) was added dropwise over 6 h. Following this, the mixture was stirred for a further 12 h at room temperature, after which 1 mL of hexane was added. The mixture was then cooled to 4 °C, and the amine hydrochloride was filtered off. The organic solvent was removed under vacuum, resulting in the formation of a yellow solid (*R*)-**83** with 87% yield (0.77 g), which was subsequently employed in the catalytic transformation with Pd precursor without further purification. For characterization of (*R*)-**83**, see ref. [72].

#### 5.2.4. Alkyl Chains as the *Linker*

**Example 10.** Synthesis of (*R*,*Sp*)-1-[1-[(6-bromohexyl)methylamino]ethyl]-2-(diphenylphosphanyl)ferrocene (**4**)

The amine (*R*,*Sp*)-**1a** (250 mg, 0.585 mmol) was dissolved in CH_3_CN (15 mL), and the resulting solution was treated with K_2_CO_3_ (84 mg, 0.614 mmol, 1.05 equiv) and 1,6-dibromohexane (178 µL, 286 mg, 1.17 mmol, 3 equiv). Subsequently, the mixture was refluxed for 2 h and then stirred at room temperature for an extension of 18 h. Thereafter, the solution was diluted with water (50 mL) and extracted with *t*-BuOMe. The combined organic extracts were subjected to a washing process with brine, followed by drying with anhydrous Na_2_SO_4_ and concentration. The crude product was purified by column chromatography on silica gel (eluent: hexane/EtOAc = 19:1, with 0.5% TEA). The purified product (*R*,*Sp*)-**4** (124 mg, 36%) was obtained as an orange oil. For characterization of (*R*,*Sp*)-**4**, see ref. [46].

**Example 11.** Synthesis of (*S*,*S*,*S*)-2-[7-bromo-2-(4,5-dihydro-4-isopropyloxazol2-yl)heptan-2-yl]-4,5-dihydro-4-isopropyloxazole (**30a**)

A flame-dried Schlenk flask was charged with (*S*,*S*,*S*)-**29a** (0.20 g, 0.79 mmol), THF (15 mL), and TMEDA (0.184 g, 0.238 mL, 1.58 mmol, 2 equiv). This was subsequently cooled to −78 °C, after which BuLi (0.32 mL, 2.5 M in hexane, 0.80 mmol, 1 equiv) was added dropwise via a syringe. The reaction mixture was then permitted to reach room temperature and stirred for a further 30 min. The solution was added dropwise via cannula to a solution of 1,5-dibromopentane (0.88 g, 3.83 mmol, 4.8 equiv) in THF (10 mL), cooled to 0 °C. Once the addition was complete, the ice bath was removed, and the solution was left to warm to room temperature and stirred for a further 16 h. Subsequently, the reaction mixture was quenched by the addition of saturated aqueous NH_4_Cl (15 mL), diluted with water (10 mL), and extracted with diethyl ether (15 mL). The organic fractions were combined, washed with brine (50 mL), and dried over MgSO_4_, and the solvent was removed under vacuum. This residue was purified by column chromatography (eluent: DCM/MeOH = 98:2) to give (*S*,*S*,*S*)-**30a** as a pale yellow oil with 68% yield (0.22 g).

The synthesis of (*S*,*S*,*S*)-2-[7-bromo-2-(4,5-dihydro-4-*tert*-butyloxazol2-yl)heptan-2-yl]-4,5-dihydro-4-*tert*-butyloxazole (**30b**) was prepared from (*S*,*S*,*S*)-**29b** with the same method, which provided a yield of 74%. For characterization of the bisoxazolines by Doherty and Knight, see ref. [57].

**Example 12.** Synthesis of (*R*,*R*)-5,5-bis(4,5-dihydro-4-phenyloxazol-2yl)nonane-1,9-di(*tert*-butyl)dimethylsilane (**33**)

A (*R*,*R*)-**32** solution (1.0 g, 3.25 mmol) in 20 mL of dry THF was cooled to 0 °C under a nitrogen atmosphere. A solution of *n*-BuLi (4.48 mL, 2.9 M in hexane, 6.50 mmol, 2 equiv) was added to the solution dropwise. The resulting mixture was stirred at 0 °C for 1 h, after which 4-iodobutoxy-(*tert*-butyl)dimethylsilane (3.68 g, 11.70 mmol, 2 equiv) was added slowly to the mixture. Once the addition was complete, the ice bath was removed, and the solution was allowed to warm to room temperature and stirred for a further 12 h. At this point, the reaction mixture was quenched by the addition of water (15 mL) and extracted with DCM. The organic fractions were combined, washed with brine and water, and then dried over Na_2_SO_4_. The solvent was removed under vacuum, and the residue was purified by column chromatography (eluent: petroleum ether/EtOAc = 10:1) to afford (*R*,*R*)-**33** as a pale yellow oil. The preparation yield of (*R*,*R*)-**33** was calculated as 70% (1.13 g). For characterization of the bisoxazolines by Zhou, see ref. [58] and the corresponding supporting information.

**Example 13.** Synthesis of (4*S*,5*S*)-4,5-bis(benzyloxymethyl)-*N*,*N’*-bis(4-chlorobutyl)imidazolidin-2-one (**97**)

A solution of 4,5-bis(benzyloxymethyl)imidazolidin-2-one (**96**, 0.6 g, 1.84 mmol) in THF (5 mL) was added to a suspension of sodium hydride (0.12 g, 95%, 4.60 mmol, 2.5 equiv) in THF (20 mL) maintained at 0 °C, and the mixture was stirred for 20 min. At this temperature, 1-bromo-4-chlorobutane (0.85 mL, 7.35 mmol, 4 equiv) was added to the reaction mixture. The reaction mixture was refluxed for 12 h. The reaction was terminated by the addition of water, and the organic compounds were extracted with DCM (15 mL × 3). The combined organic layer was subjected to a washing process involving water, brine, and drying over MgSO_4_. Following the evaporation of the solvent, the residue was purified by column chromatography on silica gel (eluent: hexane/EtOAc = 2:1), resulting in the purification of the pure product (0.85 g, 91%) as an oil. For characterization of (4*S*,5*S*)-**97**, see ref. [75].

### 5.3. Synthetic Procedures on Ionization (Quaternization) Transformation

The ionization process (also referred to as the quaternization reaction) is a pivotal step for obtaining the target ionic-liquid-supported ligands. As illustrated in the preceding synthetic strategy (Section 2.2), the ionization process could be categorized into two opposing strategies, the pre-cation approach and the pre-anion approach, in accordance with the type of reserved groups present in the parent structure. The ligands obtained through these two distinct strategies and their corresponding detailed synthetic schemes have been described in the following part. It is important to note that the purification methods employed during the ionization reaction were dependent on the solubility of the ionic liquids used, which readers may take note of while reading the following sections.

#### 5.3.1. Pre-Cation Approach

**Example 14.** Synthesis of (*R*,*R*)-1,1′-[5,5-bis(4,5-dihydro-4-phenyloxazol-2-yl)nonane-1,9]bis-(3-methyl-1*H*-imidazole) diiodide (**39**)

The solution comprising (*R*,*R*)-**37** (0.070 g, 0.13 mmol) and CH_3_I (0.145 g, 1.00 mmol, 7.7 equiv) in dry Et_2_O was stirred for 24 h at room temperature. The solvent was removed under vacuum, affording (*R*,*R*)-**39** with a yield of 86% (0.094 g). The characterization of the *C*_2_-symmetric imidazolium-loaded bisoxazoline is detailed in ref. [58].

**Example 15.** Synthesis of (*R*,*R*)-3,3′-[1-(*tert*-butoxycarbonylamino)-2-(4-dodecylphenylsulfonamido)ethane-1,2-diyl]bis(*N*,*N*,*N*-trimethylbenzenaminium) diiodide (**45b**)

The brief preparation route for (*R*,*R*)-**45** has been listed in Scheme 9. A solution of (*R*,*R*)-**44b** (5 g, 7.7 mmol) was prepared by the dissolution within 30 mL of MeOH. The solution was cooled to 0 °C using an ice bath, and formic acid (96%), with a volume of 3.7 mL (77 mmol, 10 equiv), was then added, followed by a solution of 37% formaldehyde in water (6.5 mL, 39 mmol, 5 equiv). Subsequently, the reaction mixture was maintained at reflux for overnight before being cooled to room temperature. At this stage, 100mL of water was added. Subsequently, the solution was cooled to 0 °C, and saturated aqueous Na_2_CO_3_ was added until the solution reached a pH value exceeding 10. The solution was extracted with EtOAc, and the extracts were dried over Na_2_SO_4_. The solvents were removed, resulting in the formation of a green solid.

The prepared solid was then redissolved in 30 mL of *n*-butyl alcohol. Following the addition of CH_3_I (10 mL), the solution was stirred at 80 °C for a period of 10 h. Thereafter, the solvents were removed under reduced pressure. The residue was purified by flash chromatography (eluent: DCM/MeOH) to yield a white solid (*R*,*R*)-**45b** (2.3 g, 30%). For characterization of the quaternary ammonium-salt-tagged TsDPEN ligands, see ref. [63].

**Example 16.** Synthesis of (1*R*,2*R*)-1-butyl-3-[[[2-(*tert*-butoxycarbonyl)amino-1,2-diphenylethyl]amino]methyl]pyridin-1-ium bromide (**64**)

The brief preparation route for (*R*,*R*)-**64** has been listed in Scheme 11. The synthesis (*R*,*R*)-**64** involved the reaction of the finely powdered *tert*-butyl[1,2-diphenyl-2-[(pyridin-3-yl methyl)amino]ethyl]carbamate (**63**, 753 mg, 1.8 mmol) with *n*-butyl bromide (307 mg, 2.2 mmol, 1.2 equiv) at 80 °C over 20 h. The remaining volatile materials were then removed under reduced pressure to obtain product (*R*,*R*)-**64**, which was observed to be a yellow foam (1.0 g, >99%). The obtained chiral ligand (*R*,*R*)-**64** was employed for catalytic purposes without further purification, whereas an analytical sample was purified via preparative HPLC. The characterization of the pyridinium-tagged DPEN ligands (**64**–**70**), as listed in Table 5, can be found in ref. [65].

**Example 17.** Synthesis of (1*R*,2*S*)-1-butyl-3-[[(1-hydroxy-1-phenylpropan-2-yl)amino]methyl]pyridin-1-ium bromide (**86a**)

(1*R*,2*S*)-**85** (1.14 g, 4.71 mmol) and freshly distilled *n*-butyl bromide (0.51 mL, 4.71 mmol, 1 equiv) were mixed in a sealed round-bottom flask, and the resulting mixture was stirred at 60 °C for 24 h. The excess *n*-butyl bromide was then evaporated, and the resulting brown oil was washed with anhydrous EtOAc. The remaining volatile materials were removed under reduced pressure to afford the crude product, which was purified by preparative HPLC (eluent: MeOH/H_2_O, 50:50) to give compound (1*R*,2*S*)-**86a** with 55% yield (0.89 g, 2.58 mmol). The analytical data on the pyridinium-tagged *N*,*O*-ligands are presented in Scheme 15, see ref. [73].

**Example 18.** In situ formation of (4*S*,5*S*)-Rh-complex (**102**)

A solution of bisphosphine (4*S*,5*S*)-**101** (2.8 mg, 3.7 µmol, 1.2 equiv) and [Rh(cod)_2_]BF_4_ (1.3 mg, 3.1 µmol) in DCM (1 mL) was stirred for 30 min at room temperature. Subsequently, trimethyloxonium tetrafluoroborate (1.1 mg, 7.4 µmol, 2.4 equiv) was then added, and the mixture was stirred for a period of 2 h. Evaporation of the volatiles resulted in the formation of a Rh-complex **102**. The analytical data for **102** are as follows: ^31^P NMR (121 MHz, CDCl_3_), *δ* = 32.62 (d, ^2^*J* _Rh-P_ = 139.2 Hz) [75].

#### 5.3.2. Pre-Anion Approach

**Example 19.** Synthesis of (*R*,*Rp*)-1-[2-[2-(diphenylphosphino)ferrocenyl]oxazolinyl]methyl-2,3-dimethylimidazole, trifluoromethyl sulfonate (**28a**)

A degassed solution of (*R*,*Rp*)-hydroxymethyl-2-[(2-diphenylphosphino) ferrocenyl]oxazoline (**26**, 1.5 g, 3.2 mmol) was treated with DIPEA (413 mg, 3.2 mmol, 1 equiv) in DCM (50 mL) at room temperature. Subsequently, the mixture was cooled to −40 °C, and Tf_2_O (901 mg, 3.2 mmol, 1 equiv) was introduced dropwise, maintaining the temperature for 2 h. Following this, the reaction was stirred for a further 1 h at 0 °C. The solvent was then concentrated under vacuum. The resulting crude product was immediately purified by column chromatography (eluent: petroleum ether/EtOAc = 10:1) to yield (*R*,*Rp*)-**27** as a yellow solid.

A solution of (*R*,*Rp*)-**27** in ether (75 mL) was cooled to 0 °C, after which 1,2-dimethylimidazole was added. Following a 20-min stirring, (*R*,*Rp*)-**28a** was precipitated and filtered as a yellow solid without further purification. The target product was obtained with a yield of 65% (1.45 g). For the analytical data on the prepared imidazolium-tagged oxazolinyl ferrocene ligands (**28a**–**e**), see ref. [54].

**Example 20.** Synthesis of (*S*,*S*,*S*)-3-methyl-1-[6,6-bis(4,5-dihydro-4-isopropyloxazol2-yl)heptan-1-yl]imidazolium bromide (**31a**)

A solution of bisoxazoline (*S*,*S*,*S*)-**30a** (0.66 g, 1.64 mmol) and 2-methylimidazole (0.270 g, 3.28 mmol, 2 equiv) in toluene (5 mL) was heated at 100 °C for 16 h. Thereafter, the solution was cooled to room temperature and the solvent removed under vacuum. Subsequently, the resulting residue was subjected to exhaustive washing with hexane to remove the residual 2-methylimidazole, after which purification was achieved through the gradual addition of hexane to a dichloromethane solution. This process afforded (*S*,*S*,*S*)-**31a** as a spectroscopically and analytically pure colorless oil, with an overall yield of 94% (0.62 g).

A similar chiral bisoxazoline ligand (*S*,*S*,*S*)-**31b** was obtained using the same preparative process. The characterizations of the imidazolium-supported bisoxazolines were presented in ref. [57].

**Example 21.** Synthesis of (*R*,*R*)-1,1′-[5,5-bis(4,5-dihydro-4-phenyloxazol-2yl)nonane-1,9]bis-(1,2-dimethyl-1*H*-imidazole) diOTs (**38a**)

(*R*,*R*)-5,5-bis(4,5-dihydro-4-phenyloxazol-2yl)nonane-1,9-ditosylate (**35**, 0.270 g, 0.36 mmol) and 1,2-dimethyl-1*H*-imidazole were combined and dissolved in 2 mL of DMF. The solution was then heated to 70 °C for 24 h under a nitrogen atmosphere. The solvent DMF was then removed under vacuum after the completion of the quaternization. The resulting residue was then washed with Et_2_O until a light-yellow solid was observed. The target *C*_2_-symmetric imidazolium-loaded bisoxazoline **38a** was obtained in 85% yield (0.29 g) with analytical data presented in ref. [58].

**Example 22.** Synthesis of (*S*,*S*)-1-[4-[4-[[[2-[[(1,1-dimethylethoxy)carbonyl]amino]-1,2-diphenylethyl]amino]sulfonyl]phenoxy]butyl]3-methyl-1*H*-imidazolium chloride (**50**)

A mixture of (*S*,*S*)-*N*-[2-[(1,1-dimethylethoxy)carbonyl]amino-1,2-diphenylethyl]-4-(4-chlorobutoxy)benzene sulfonamide (**49**, 117 mg, 0.209 mmol) and 1-methylimidazole (0.167 mL, 2.09 mmol, 10 equiv) was heated at 88 °C for 11 h under a nitrogen atmosphere. The volatile material was removed under reduced pressure, and the residue was subjected to a washing process with EtOAc in order to obtain the compound (*S*,*S*)-**50** with a yield of 95% (128 mg).

The ionic-liquid-supported DPEN ligands (**50**–**55**) provided in Table 4 were prepared in the same manner. For the analytical data concerning the synthesized supported DPEN ligands, see ref. [64].

### 5.4. Ion Exchange Strategies of Ionic-Liquid-Supported Ligands and Blocks

The composition of the ionic fragments presented in the ionic-liquid-supported ligands and catalysts has a significant influence on the yield and enantioselectivity of the chiral products generated during catalyzed reactions, in addition to the feasibility of recovering the ligand for recycling purposes. This finding has been corroborated by the catalytic results, such as (*R*,*Sp*)-**12** and (*R*,*R*)-**74**. As a means of enhancing the ligand structural diversity, anion-exchange strategies have been employed in the synthesis of select ligands.

#### 5.4.1. Ion Exchange Based on Ionic-Liquid-Supported Ligands

**Example 23.** Synthesis of (*R*,*Rp*)-1-[2-[2-(diphenylphosphino)ferrocenyl]oxazolinyl]methyl-2,3-dimethylimidazole, hexafluorophosphate (**28b**)

Saturated aqueous KPF_6_ (250 mg, 1.36 mmol, 10 equiv) was introduced to a solution of (*R*,*Rp*)-1-[2-[2-(diphenylphosphino)ferrocenyl]oxazolinyl]methyl-2,3-dimethylimidazole, trifluoromethyl sulfonate (*R*,*Rp*)-**28a** (100 mg, 0.14 mmol), which had been degassed prior to use. Following a period of stirring for one hour, (*R*,*Rp*)-**28b** was precipitated and filtered as a yellow solid, having been produced in a yield of 88% (87 mg). No further purification was necessary at this stage. The desired product (*R*,*Rp*)-**28b**, with the modified anion, was obtained with a yield of 88% (87 mg) [54].

**Example 24.** Synthesis of (*S*,*S*,*S*)-3-Methyl-1-[6,6-bis(4,5-dihydro-4-tert-butyloxazol2-yl)heptan-1-yl]imidazolium bis[(trifluoromethyl)sulfonyl]imide (**31c**)

(*S*,*S*,*S*)-**31b** (0.400 g, 0.08 mmol) was dissolved in DCM (10 mL) and treated with a solution of LiNTf_2_ (0.024 g, 0.084 mmol, 1.05 equiv) in water (5 mL). The resulting mixture was stirred vigorously for 6 h, after which the organic layer was separated and successively washed with water. The solvent was removed, and the resulting liquid was then subjected to a four-hour drying process at 60 °C with 0.1 mbar of pressure, which afforded (*S*,*S*,*S*)-**31c** free of Br (without the formation of any precipitate with AgNO_3_). The bisoxazoline ligand with NTf_2_ anion was obtained with 96% yield (0.531 g) [57].

#### 5.4.2. Ion Exchange Based on Ionic-Liquid-Loaded Blocks

**Scheme 19:**
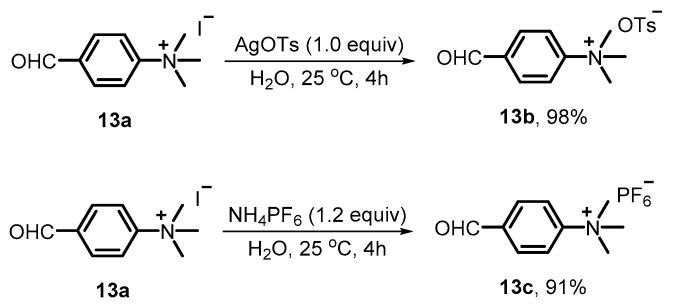
Anion exchange form 4-formyl-*N*,*N*,*N*-trimethyl-benzenaminium iodide.

The synthetic methods presented in the following examples are illustrated in Scheme 19. The analytical data for the prepared ionic-liquid-loaded blocks were reported in ref. [50].

**Example 25.** Synthesis of 4-formyl-*N*,*N*,*N*-trimethyl-benzenaminium toluenesulfonate (**13b**).

A solution of 4-formyl-*N*,*N*,*N*-trimethyl-benzenaminium iodide (**13a**, 1.455 g, 5 mmol) in water (5 mL) was prepared, and AgOTs (1.395 g, 5 mmol, 1 equiv) was added to this solution. The solution was agitated for a period of 4 h at 25 °C. Upon completion of the reaction, the AgI precipitate was isolated by filtration. The filtrate was concentrated under reduced pressure. The resulting white solid **13b** was then dried under vacuum, yielding a yield of 98%.

**Example 26.** Synthesis of 4-formyl-*N*,*N*,*N*-trimethyl-benzenaminium hexafluorophosphate (**13c**).

A solution of 4-formyl-*N*,*N*,*N*-trimethyl-benzenaminium iodide (**13a**, 1.455 g, 5 mmol) in water (5 mL) was prepared, and then NH_4_PF_6_ (984 mg, 6 mmol, 1.2 equiv) was added. The solution was agitated for a period of 4 h at 25 °C. Upon completion of the reaction, the 4-formyl-*N*,*N*,*N*-trimethyl-benzenaminium hexafluorophosphate (**13c**) was observed to precipitate from the solution. The precipitate was isolated by filtration, washed with water, and then dried under vacuum. The yield of **13c** was calculated as 91%.

## 6. Summary and Outlook

The combination of traditional chiral organic ligands with ionic liquids represents a highly innovative approach that expands the utility of non-supported transition-metal chiral catalysts. These catalysts, known for their exceptional stereocontrol in asymmetric catalysis, can be transitioned from organic systems to environmentally friendly reaction media (such as ionic liquids and water), enabling their use not only in the catalytic synthesis of optically active molecules with excellent stereoselectivity but also in the recycling of difficult-to-recover transition-metal catalysts. In this brief review, we aim to categorize the literature on chiral ionic-liquid-supported ligands that have been synthesized and successfully applied in transition-metal-catalyzed reactions. Unlike the previously published reviews, which primarily focus on the outcomes of these functionalized catalysts in the production of optically active compounds, our work emphasizes the elucidation of the structures of these supported ligands and the corresponding synthesis strategies.

The ionic liquid moieties, which are crucial components of the ligands discussed, are typically incorporated in a traceable manner during the synthetic processes of the ligands. In Section 2 of this manuscript, we provide a detailed overview of the strategies for integrating ionic liquid fragments into traditional chiral ligands. We then categorize the parent structures of these chiral ligands in Section 3, offering a coherent explanation of the overall synthetic routes for chiral ionic-liquid-supported ligands. In Section 4, we list the transition-metal precursors used in conjunction with these ligands for asymmetric catalytic reactions. These transition-metal precursors, in combination with chiral ligands featuring various backbones and coordination groups, have been employed in a wide range of catalytic reactions with different mechanisms, aimed at producing chiral molecules with high stereoselectivity.

It is worth noting that the key steps in the synthetic routes presented in Section 3 can be challenging to fully illustrate through complete reaction pathways. Therefore, in Section 5, we explore selected synthetic schemes to help readers focus on the critical aspects and potential challenges encountered during the synthesis process.

In recent years, particularly in the past decade, there have been relatively few reports on the synthesis and practical application of ionic-liquid-supported catalysts, specifically chiral organic ligands for transition-metal complexes. This suggests that the process of modifying ligands with ionic liquids may still have notable limitations compared to traditional ligands and transition-metal catalysts. We posit that chemists could expand the scope of applications for ionic liquid-loaded chiral ligands and their corresponding catalysts and achieve breakthrough results through further in-depth research in the following areas.

### 6.1. Facile Strategies for the Synthesis of Chiral Ionic-Liquid-Supported Ligands

The great diversity of chiral ionic-liquid-supported ligands arises from variations in ligand scaffolds, ionic liquid substructures, and the integration sites of functional groups within these scaffolds. This diversity has, in turn, driven the development of sophisticated synthetic methodologies specifically tailored for the synthesis of ionic-liquid-supported ligands. However, the broad range of synthetic strategies also highlights the need for innovative approaches in the development of new ligand variants. Consequently, these specialized methods have hindered the widespread adoption and application of such ligands and their corresponding transition-metal catalysts. The development of simplified methodologies that can be applied to various ligand parent structures and ionic liquid fragments remains a key topic in this field.

In organic synthesis, the concept of modular synthesis has been successfully employed in the production of a diverse array of significant substances, including bioactive molecules [76,77], natural products [78], polymers [79], and materials [80]. In chiral catalysis, modular synthesis has already been applied in the design and synthesis of Salen catalysts and oxazolidine ligands by Weck [81] and Wolf [82], respectively. Given the encouraging results from other areas of research, we propose that a modular approach to the synthesis of ionic-liquid-supported compounds, particularly chiral ligands, represents an exciting avenue for investigation in this field.

The modular synthetic design strategy allows for the creation of new chiral ligands and catalysts, enhancing their catalytic efficiency and ensuring precise yields of stereoselective organic products. Additionally, this approach provides a solid foundation for the large-scale production and industrial application of supported chiral catalysts in the future.

### 6.2. Reliable Recycling Programs for Immobilized Transition-Metal Catalysts

The immobilized transition-metal catalysts discussed in this review consistently exhibited a significant decline in performance after fewer than ten recycling cycles, both in homogeneous and heterogeneous reaction systems. This decline in performance was primarily attributed to the dissociation of chiral ligands from the transition-metal complexes, which led to structural degradation and subsequent catalyst deactivation [54,71,75,83]. Such dissociation severely hindered the preparation efficiency of optically active products, particularly affecting their stereoselectivity. Another major contributing factor was catalyst leaching during the recycling process [46,52,57]. Due to the inherent limitations of the recycling procedure, ionic-liquid-supported transition-metal catalysts typically experienced minor losses during each cycle. As the number of cycles increased, the cumulative loss resulted in a reduction of the active catalyst concentration within the system, ultimately contributing to the diminished catalytic capacity of these transition-metal complex catalysts.

The development of reliable recovery techniques for transition-metal chiral catalysts, particularly those incorporating ionic-liquid-functionalized segments, is crucial for efficient catalytic applications and recycling.

(1) Structurally robust chiral ionic-liquid-supported ligands and catalysts

The structural stability of catalysts has been demonstrated to be a key factor in enhancing their effective recyclability. It is reasonable to anticipate that in the near future, researchers will develop chiral ionic-liquid-supported ligands and catalysts with high turnover numbers (TON) and turnover frequencies (TOF), alongside excellent stereoselective catalytic performance. However, the structural instability of charged organic compounds and the potential for structural alterations in transition-metal complexes during extended catalytic processes pose significant challenges to maintaining the integrity of ionic-liquid-supported catalysts.

(2) Dependable catalyst recycling protocols in batch reaction systems

One of the most compelling features of ionic-liquid-supported catalysts is their excellent solubility in hydrophilic solvents, such as hydrophilic ionic liquids. This characteristic allows for convenient separation of hydrophobic organic products from the hydrophilic catalyst in batch reaction systems. However, it is important to note that recovery strategies, recovery frequency, and the stability of catalytic results can vary significantly depending on the structural differences of the supported catalysts and their application contexts, as reported in the literature. As a result, the development of reliable recycling techniques tailored to environmentally sensitive transition-metal catalysts in batch reaction systems has become a critical topic in this research field.

The utilization and practical recycling methodologies of chiral ionic-liquid-supported ligands and their corresponding transition-metal catalysts are currently being investigated in our group.

### 6.3. Economical Industrial-Scale Application Precedents

Numerous examples of industrial-scale reactions utilizing conventional transition-metal catalysts within traditional organic synthesis frameworks have been demonstrated over the past few decades [84,85,86,87,88,89]. Furthermore, the use of ionic liquid systems has gained traction in industrial applications in recent years [90,91,92,93]. However, instances of industrial-scale deployment of chiral ligands with ionic liquid fragments and their corresponding transition-metal complex catalysts remain limited.

The reasons for this application limitation are manifold. The high costs associated with the complex synthesis routes for preparing the supported ligands and corresponding catalysts, as well as the elevated prices of environmentally friendly reaction systems (e.g., ionic liquids), directly contribute to a significant increase in the manufacturing costs of chiral organic compounds. This makes the profitability of producing chiral organic molecules under such conditions considerably diminished. These challenges in chiral catalytic design have made it difficult to convince industrial producers to pursue further research and investment in this area.

Therefore, despite being labeled as environmentally friendly or chemically green, further in-depth exploration by researchers is required regarding the application of chiral ionic-liquid-supported transition-metal catalysis in industrial-scale manufacturing.

### 6.4. Systematic Description of Charge-Containing Fragments on the Mechanism of Recyclable Stereoselective Transformations

In specific examples, the stereoselectivity, particularly enantioselectivity, of organic molecules produced using ionic-liquid-supported catalysts was found to be slightly enhanced compared to conventional transition-metal catalysts in similar catalytic reaction processes [50,54,55,57,71,75]. This phenomenon suggests that the incorporation of an ionic liquid moiety contributes to improved product selectivity under comparable reaction conditions. In these studies, researchers have explored the impact of charged ionic liquid fragments on the yield and stereoselectivity of optically active products during asymmetric reactions, offering valuable insights. However, the current conclusions do not fully address the role of the charged fragments throughout the entire catalytic cycle.

Further investigation is needed to deepen the understanding of the mechanism behind asymmetric transformations involving ionic-liquid-supported ligands, with particular focus on the following outstanding issues:

(1) Effects of charge-containing fragments on ligand structure and transition metal complexes

The current studies provide only limited conclusions regarding the effect of charge-containing fragments on the structure of ligands and the corresponding transition-metal complexes. In 2019, Bica investigated the impact of ionic fragments on the backbone of modified DPEN ligands using DFT calculations [65]. The results showed that there were no significant intramolecular interactions between the ionic fragments and the DPEN skeleton. Similarly, the alteration of the negative molecular electrostatic potential of the Boc group in the ligand was found to be minimal.

Notably, the authors of the original work also emphasized the need for additional methods to evaluate the overall structure of the catalyst. Indeed, the structural impact of charge-bearing fragments on catalysts remains a relatively underexplored area. A deeper understanding of this effect will provide a crucial foundation for the development of highly efficient catalysts.

(2) Effects of charge-containing fragments on key reaction intermediates

The role of ionic liquid fragments incorporated into chiral ligand structures extends beyond the contribution of the functional group itself. In certain applications, the effects of charge-containing fragments on the catalytic reaction rate and the stereoselectivity of the optically active product have been observed. Jing and colleagues demonstrated that the catalytic activity in the asymmetric cycloaddition of racemic propylene oxide and CO_2_ varied with different axial anions [70]. Additionally, the enantioselectivity of the resulting chiral cyclic carbonate showed differential outcomes depending on the presence of the axial anions.

In another example, Zhou’s group showed that both the cations and anions in the charged fragments positively influenced the stereoselectivity of chiral cycloaddition products, as indicated by DFT calculations [54]. The calculations revealed that the positively charged imidazolium moiety imparted more rigid steric hindrance to the azomethine ylides, thereby enhancing the stereoselectivity of the desired cycloaddition products. This hypothesis was later confirmed by experimental catalytic results.

Similar findings were reported in applications involving ionic-liquid-supported organocatalysts. Headley and colleagues explored the potential of pyridinium-tagged pyrrolidines as chiral catalysts for asymmetric Michael addition reactions, obtaining target products with excellent enantioselectivity [94]. The authors suggested that the interaction between the pyridinium cation and the nitro group of the substrate played a beneficial role in the observed stereoselectivity.

The influence of charge-containing fragments on the selectivity of chiral products is clearly evident from these examples. However, it is apparent that the current discussion on the effect of charge-containing groups on key intermediates remains limited. We contend that this significant mode of action warrants further investigation and attention from researchers in the field. We anticipate that the results of future studies will provide a solid theoretical foundation for the broader application of ionic-liquid-supported catalysts.

(3) Interactions between charge-containing fragments and solvents.

Ionic-liquid-supported catalysts offer a reliable framework for reaction systems using water as a solvent. Chiral DPEN ligands with hydrophilic charged fragments were employed in the reduction of aliphatic ketones through asymmetric reduction, as demonstrated in Deng’s work [63]. EM analysis revealed that the chiral catalysts formed metal micelles in water, with the reaction occurring within these micelles.

Similarly, Zlotin presented an ionic-liquid-supported organocatalyst for asymmetric aldol reactions in the presence of water [95]. The unusual hydrogen-bonding ability of the ionic liquid moiety with substrates in the aqueous phase was identified as a key factor contributing to the high enantioselectivity observed in the reactions.

It is clear that the charge-containing fragments within the catalyst act as bridges, connecting the hydrophobic organic substrate to the solvent (water), as exemplified in these intriguing applications. A deeper understanding of the interactions between charge-containing fragments and solvents would greatly enhance the rational design, synthesis, and application of advanced catalysts for the efficient production of highly enantio-enriched building blocks in environmentally friendly systems.

## Figures and Tables

**Table 1:** Selected linker and the structure of presentative chiral ligands/catalysts.

Entry	*Linker*	Selected Ligands/Catalysts	
1	amides and sulfonamides	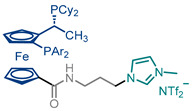	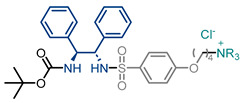
2	imines	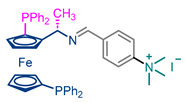	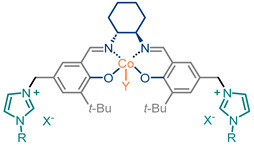
3	phosphite	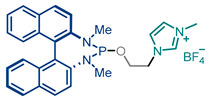	
4	alkyl chains	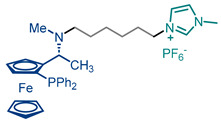	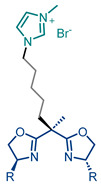
		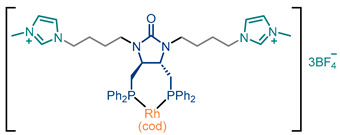	

**Table 2:** Structure and preparation yield of quaternary-ammonium-tagged ferrocene ligands.

Entry	Compound No.	Parent Ligand	Ionic Liquid Moiety	Preparation Yield
1	(*R*,*Sp*)-**15**	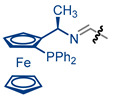	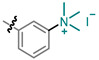	90%
2	(*R*,*Sp*)-**16**		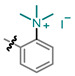	95%
3	(*R*,*Sp*)-**17**		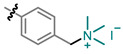	93%
4	(*R*,*Sp*)-**18**		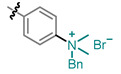	96%

**Table 3:** Structure and preparation yield of quaternary-ammonium-tagged diphosphine ferrocene ligands.

Entry	Compound No.	Parent Ligand	Ionic Liquid Moiety	Reaction Condition	Preparation Yield
1	(*R*,*Sp*)-**21**	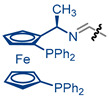	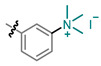	Condition A	96%
2	(*R*,*Sp*)-**22**		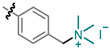	Condition A	94%
3	(*R*,*Sp*)-**23**		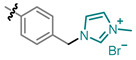	Condition A	95%
4	(*R*,*Sp*)-**24**		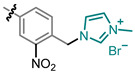	Condition C: EtOH:DCM = 1:1, reflux, 2h	95%
5	(*R*,*Sp*)-**25**		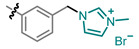	Condition D: DCM, reflux, 3 h	95%

**Table 4:** Structure of *C*_2_-symmetric ionic-liquid-supported bisoxazolines reported by Zhou.

Entry	Compound No.	Ligand Backbone	Counterpart Anion
1	(*S*,*S*)-**40a**	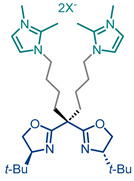	X = OTs
2	(*S*,*S*)-**40b**		X = PF_6_
3	(*S*,*R*,*S’*,*R’*)-**41**	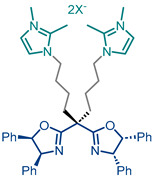	X = OTs
4	(*S*,*S*)-**42a**	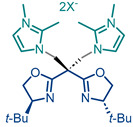	X = OTs
5	(*S*,*S*)-**42b**		X = PF_6_

**Table 5:** List of ionic-liquid-supported TsDPEN quaternary ammoniums with chloride anion.

Entry	Compound No.	Ligand Backbone	Ionic Liquid Moiety	Solvent	Preparation Yield
1	(*S*,*S*)-**50**	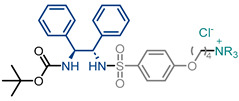	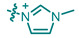	None	95%
2	(*S*,*S*)-**51**		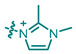	None	95%
3	(*S*,*S*)-**52**		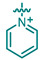	None	99%
4	(*S*,*S*)-**53**		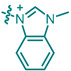	CH_3_CN	82%
5	(*S*,*S*)-**54**		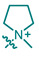	None	56%
6	(*S*,*S*)-**55**		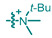	CH_3_CN	86%

**Table 6:** List of ionic-liquid-supported DPEN quaternary ammoniums with trifluoroacetic acid.

Entry	Compound No.	Ligand Backbone	Ionic Liquid Moiety	Solvent	Preparation Yield
1	(*S*,*S*)-**56**	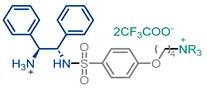	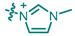	None	99%
2	(*S*,*S*)-**57**		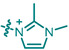	None	95%
3	(*S*,*S*)-**58**		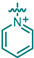	None	96%
4	(*S*,*S*)-**59**		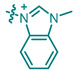	CH_3_CN	99%
5	(*S*,*S*)-**60**		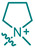	None	92%
6	(*S*,*S*)-**61**		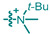	CH_3_CN	99%

**Table 7:** Structure and preparation yield of pyridinium-tagged DPEN ligands in Bica’s work.

Entry	Compound No.	Ligand Backbone	R	n	Preparation Yield (Quaternization Step)
1	(*R*,*R*)-**65**	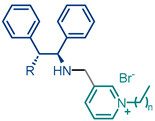	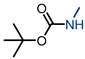	12	99%
2	(*R*,*R*)-**66**		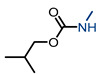	4	99%
3	(*R*,*R*)-**67**		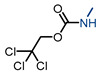	4	95%
4	(*R*,*R*)-**68**		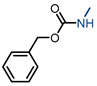	4	99%
5	(*R*,*R*)-**69**		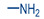	4	34%
6	(*R*,*R*)-**70**		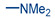	4	88%

**Table 8:** Selected structure of imidazolium-tagged Salen-Co(III)-Y complexes for the asymmetric cycloaddition to epoxides.

Entry	Compound No.	Ligand Backbone	R	X	Y
1	(*R*,*R*)-**74a**	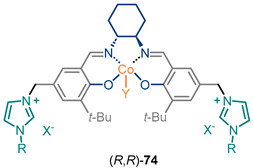	butyl	Br	OAc
2	(*R*,*R*)-**74b**		octyl	Br	OAc
3	(*R*,*R*)-**74c**		dodecyl	Br	OAc
4	(*R*,*R*)-**74d**		butyl	Cl	OAc
5	(*R*,*R*)-**74e**		octyl	Cl	OAc
6	(*R*,*R*)-**74f**		dodecyl	Cl	OAc
7	(*R*,*R*)-**74g**		dodecyl	Cl	CF_3_COO
8	(*R*,*R*)-**74h**		dodecyl	Cl	CCl_3_COO
9	(*R*,*R*)-**74i**		dodecyl	Cl	OTs

**Table 9:** Structure of chiral Schiff base ligands.

Entry	Compound No.	Ligand Backbone	m	n
1	(*R*)-**95a**	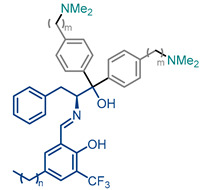	1	11
2	(*R*)-**95b**		1	−1
3	(*R*)-**95c**		2	−1
4	(*R*)-**95d**		2	7
5	(*R*)-**95e**		2	11

**Table 10:** Transition-metal precursors and corresponding chiral ionic-liquid-supported ligands (catalysts).

Entry	Type of Transition Metal	Transition-Metal Precursors	Ionic-Liquid-Supported Ligands *^a^*	Ref.
1	Palladium (Pd)	[Pd(allyl)Cl]_2_	**3**, **5**, **8**; **14**; **83**	[46,50,72]
		[Pd_2_(dba)_3_]·CHCl_3_	**3**, **5**, **8**	[46]
		Pd(CH_3_CN)_2_Cl_2_	**20**	[52]
2	Rhodium (Rh)	[Rh(NBD)_2_]BF_4_	**11**	[49]
		[Cp*RhCl_2_]_2_	**46**	[63]
		[Rh(COD)_2_]BF_4_	**101**	[75]
3	Ruthenium (Ru)	[RuCl_2_(benzene)]_2_	**56–61**	[64]
		Ru[(*p*-cymene)Cl_2_]_2_	**64–70**; **86**, **89**, **92**	[65,73]
		Ru[(2-methylallyl)_2_(COD)]	**78**, **79**	[71]
4	Copper (Cu)	Cu(CH_3_CN)_4_ClO_4_	**28**	[54]
		Cu(OTf)_2_	**31**; **38-42**	[57,58]
5	Cobalt (Co)	Co(OAc)_2_·H_2_O	**74**	[70]
6	Zinc (Zn)	Zn(NO_3_)_2_	**95**	[74]

*^a^* the labels of stereo-configuration of the presented ligands are omitted in the table.

## Data Availability

No new data were created or analyzed in this study. Data sharing is not applicable to this article.
